# The *Ascaris suum* nicotinic receptor, ACR-16, as a drug target: Four novel negative allosteric modulators from virtual screening

**DOI:** 10.1016/j.ijpddr.2016.02.001

**Published:** 2016-02-10

**Authors:** Fudan Zheng, Alan P. Robertson, Melanie Abongwa, Edward W. Yu, Richard J. Martin

**Affiliations:** a Department of Chemistry, College of Liberal Arts and Sciences, Iowa State University, Ames, IA 50011, USA; b Department of Biomedical Sciences, College of Veterinary Medicine, Iowa State University, Ames, IA 50011, USA; c Department of Physics and Astronomy, College of Liberal Arts and Sciences, Iowa State University, Ames, IA 50011, USA

**Keywords:** Asu-ACR-16, Structure-based drug discovery, Homology modeling, Orthosteric site, Allosteric modulator, *Xenopus* expression, ECD, extracellular domain, TID, transmembrane and intracellular domain, (+), principal subunit, (−), complementary subunit, NAM, negative allosteric modulator, nAChR, nicotinic acetylcholine receptor, AChBP, acetylcholine-binding protein

## Abstract

Soil-transmitted helminth infections in humans and livestock cause significant debility, reduced productivity and economic losses globally. There are a limited number of effective anthelmintic drugs available for treating helminths infections, and their frequent use has led to the development of resistance in many parasite species. There is an urgent need for novel therapeutic drugs for treating these parasites. We have chosen the ACR-16 nicotinic acetylcholine receptor of *Ascaris suum* (Asu-ACR-16), as a drug target and have developed three-dimensional models of this transmembrane protein receptor to facilitate the search for new bioactive compounds. Using the human α7 nAChR chimeras and *Torpedo marmorata* nAChR for homology modeling, we defined orthosteric and allosteric binding sites on the Asu-ACR-16 receptor for virtual screening. We identified four ligands that bind to sites on Asu-ACR-16 and tested their activity using electrophysiological recording from Asu-ACR-16 receptors expressed in *Xenopus* oocytes. The four ligands were acetylcholine inhibitors (SB-277011-A, IC_50_, 3.12 ± 1.29 μM; (+)-butaclamol Cl, IC_50_, 9.85 ± 2.37 μM; fmoc-1, IC_50_, 10.00 ± 1.38 μM; fmoc-2, IC_50_, 16.67 ± 1.95 μM) that behaved like negative allosteric modulators. Our work illustrates a structure-based in silico screening method for seeking anthelmintic hits, which can then be tested electrophysiologically for further characterization.

## Highlights

• Three-dimensional structural models of the *Ascaris* nicotinic (Asu-ACR-16) receptor made by homology modeling.
• High affinity ligands selected by *in silico* screening.
• Four ligands validated by electrophysiological studies as negative allosteric modulators.

## Graphical abstract

**Figure:**
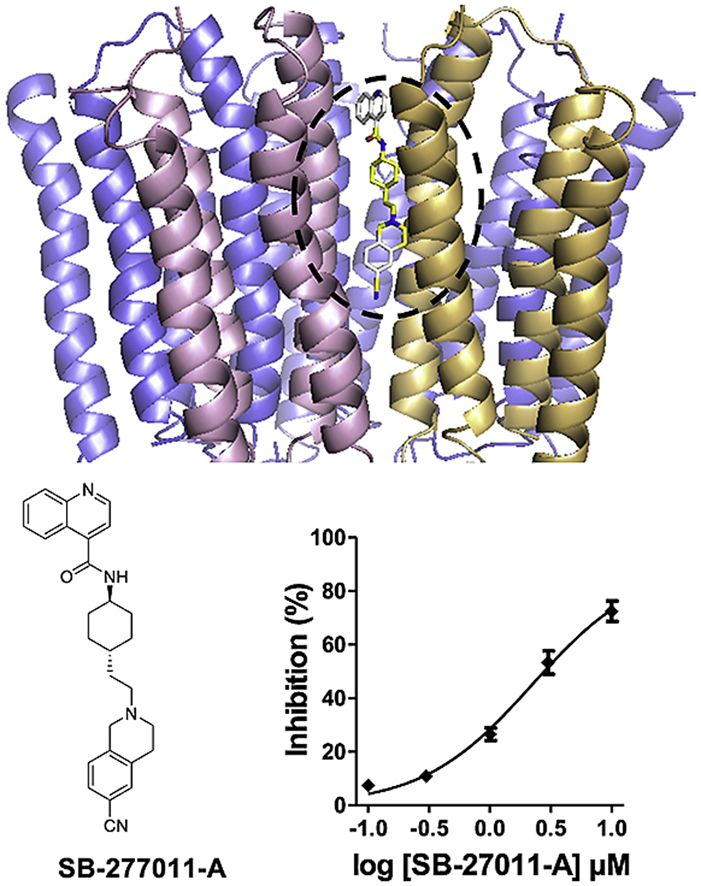

## 1 Introduction

Soil-transmitted gastrointestinal nematodes, namely roundworms, whipworms and hookworms, infect approximately two billion people worldwide and pose a significant health challenge to humans and animals (de Silva et al., 2003, Bethony et al., 2006). The infections with the soil-transmitted helminths can cause malnutrition, iron-deficiency anemia and impaired cognitive performance (Crompton, 2000, Hotez et al., 2007). Currently, there are no effective vaccines available (Hewitson and Maizels, 2014), and sanitation is not adequate in many countries. The World Health Organization (WHO) recommends four anthelmintics for treatment and prophylaxis of soil-transmitted nematode infections: albendazole, mebendazole, levamisole and pyrantel (Keiser and Utzinger, 2008). The repeated use of a limited number of anthelmintic drugs has led to an increase in drug resistance in animals and there are similar concerns for humans. It is therefore important to identify novel therapeutic compounds that selectively target receptors of parasitic nematodes so that we maintain effective therapeutics.

The nicotinic acetylcholine receptors (nAChRs) are pentameric ligand-gated ion channels that mediate synaptic transmission at neuromuscular junctions of vertebrates and invertebrates (Changeux and Edelstein, 1998). The neurotransmitter, acetylcholine, activates nAChRs by binding to orthosteric binding sites on the extracellular domain of the receptor and triggers the opening of the channel pore in the transmembrane domain. The opening of the nicotinic receptors leads to an influx of sodium and calcium depending on the receptor subtypes, as well as an output of potassium ions, followed by membrane depolarization and muscle contraction.

Nicotinic anthelmintics are selective agonists of nematode muscle nAChRs which cause spastic paralysis of the parasites (Martin and Robertson, 2010, Buxton et al., 2014). There are three different pharmacological subtypes of nAChRs present on muscle of *Ascaris suum*. The anthelmintics, levamisole and pyrantel are selective agonists of L-subtypes of nAChRs in *A. suum* (Martin et al., 2012). Bephenium selectively activates B-subtypes of nAChRs. Nicotine and oxantel selectively activate N-subtypes of nAChRs in *A. suum* (Qian et al., 2006). The anthelmintic monepantel activates nAChRs which are composed of DEG-3-like subunits (*Haemonchus. contortus* MPTL-1, *Caenorhabditis. elegans* ACR-20 and *H. contortus* ACR-23 subunits (Rufener et al., 2010, Buxton et al., 2014). We have selected the N-subtype of nAChR that is composed of ACR-16 subunits (Ballivet et al., 1996, Polli et al., 2015) for a drug target, because it is pharmacologically different to the other nicotinic receptor subtypes (Raymond et al., 2000), for further study. Asu-ACR-16 transcript has been found in *A. suum* muscle and may be involved in locomotion.

The ACR-16 nicotinic acetylcholine receptor of *A. suum* (Asu-ACR-16) is a homomeric receptor made up of five identical α subunits. Homomeric nAChRs have five identical orthosteric binding sites where agonists and competitive antagonists bind at the interface of two adjacent subunits. The orthosteric site is in the extracellular domain and is formed by the loops A, B & C of the principal subunit and by the loops D, E & F on the complementary subunit (Galzi et al., 1991, Arias, 2000). In addition, three allosteric binding sites close to the orthosteric binding sites in the extracellular domain have been observed in the α7 nAChR-AChBP chimera (Spurny et al., 2015). In the transmembrane domain, an intrasubunit allosteric binding site has been found in *Rattus. norvegicus* α7 nAChR (Young et al., 2008), while an intersubunit allosteric binding site has been found in C. elegans glutamate-gated chloride channel (GluCl) (Hibbs and Gouaux, 2011). These well-studied binding sites in nAChRs or other Cys-loop receptors provided our framework for characterizing putative orthosteric and allosteric sites in Asu-ACR-16.

Because of the lack of a crystal structure for Asu-ACR-16, we used homology modeling to predict the protein structure, based on the observations that proteins with similar sequences usually have similar structures (Cavasotto and Phatak, 2009). In this study, we used homology modeling to predict the three-dimensional structure of Asu-ACR-16, based on the observed experimental structures of the human α7 nAChR chimeras and the *Torpedo marmorata* nAChR as templates. Virtual screening was performed for the ACR-16 orthosteric binding sites, using the predicted structure to identify the potential candidates of agonists and competitive antagonists. Allosteric binding sites were also used to examine the binding properties of the virtual screening hits. Subsequently, we tested the pharmacological profiles of virtual screening hits on Asu-ACR-16 receptors expressed in *Xenopus laevis* oocytes, using a two-electrode voltage clamp to test the activity of the hits on the receptors.

## 2 Materials and methods

### 2.1 Identification of template structures

We selected the extracellular domain of Asu-ACR-16 (ECD-Asu-ACR-16) because it forms a homologomer that allows homology modeling. In addition, many of the agonists that activate Asu-ACR-16, acetylcholine, nicotine, cytisine, epibatidine (Abongwa et al., 2016), are also known to bind to the orthosteric binding sites of extracellular domain of *Lymnaea stagnalis* AChBP or *A. california* AChBP (Celie et al., 2004, Li et al., 2011, Rucktooa et al., 2012, Olsen et al., 2014a). In addition to the orthosteric binding site, three separate allosteric binding sites in the extracellular domain of α7 nAChR are now recognized (Bertrand et al., 2008, Pan et al., 2012, Spurny et al., 2015), increasing the possibility of identifying allosteric modulators.

**Fig. 1:**
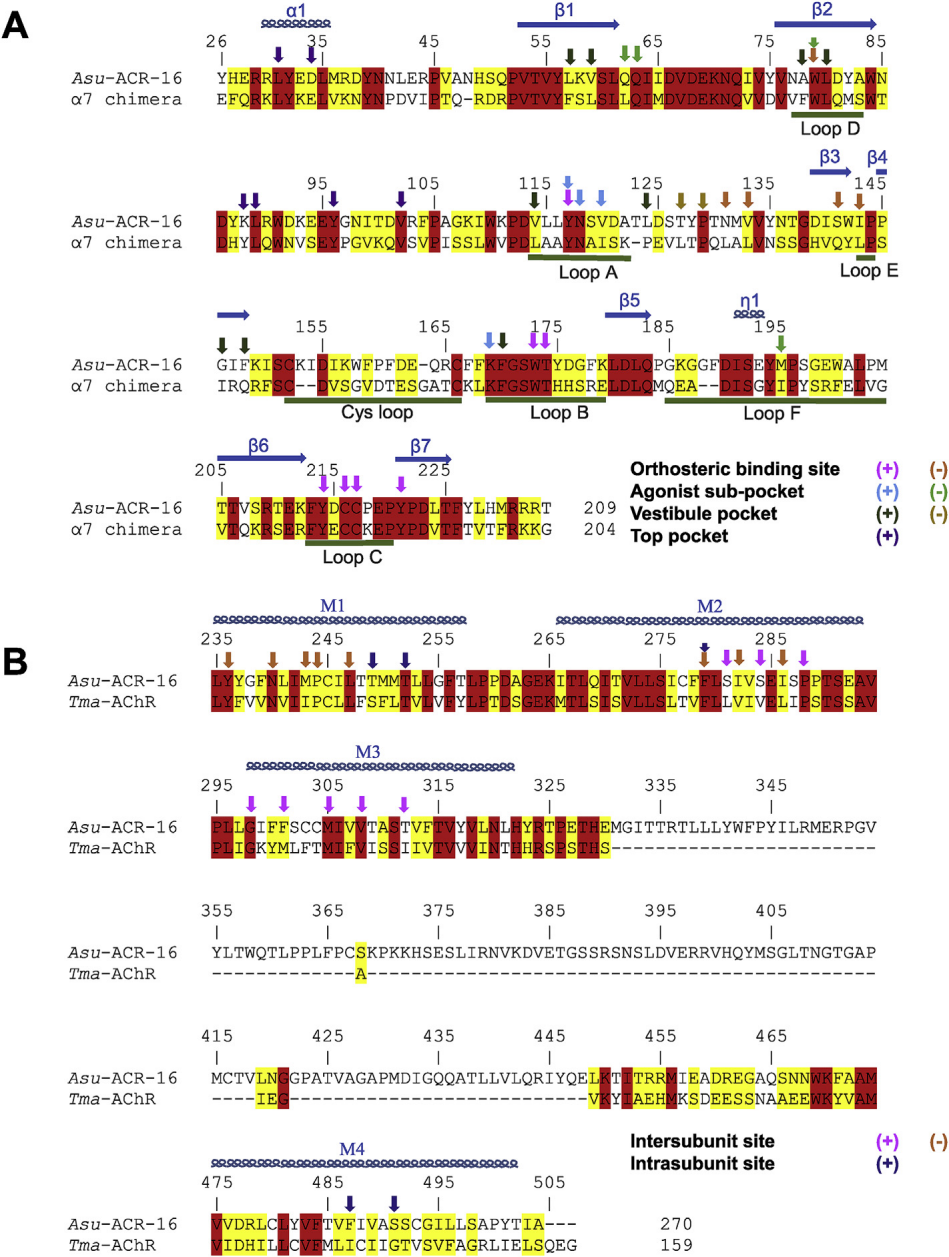
(A) Sequence and numbering of the ECD-Asu-ACR-16 and its alignment with the template, human α_7_ nAChR chimera subunit. Completely conserved residues (red background) and partially conserved residues (yellow background) are indicated. Secondary structures are shown schematically above the sequences. α_1_ represents α helix. β_1-7_ represent β sheet. η_1_ represents α helix. The Cysteine loop and loops A - F are labeled by dark green bars. Residues in the orthosteric binding site are indicated by arrows (principal subunit, pink; complementary subunit, orange). Residues in three allosteric binding pockets are highlighted by arrows (principal subunit of agonist sub-pocket, turquoise; complementary subunit of agonist sub-pocket, green; principal subunit of vestibule pocket, dark green; complementary subunit of vestibule pocket, gold; principal subunit of top pocket, purple). (B) Sequence and numbering of the TID-Asu-ACR-16 and its alignment with the template TID-Tma-AChR subunit A. Completely conserved residues (red background) and partially conserved residues (yellow background) are indicated. Four transmembrane α helixes (M1, M2, M3 and M4) are shown schematically above the sequences. Residues in the allosteric binding pocket are indicated by arrows (principal subunit, pink; complementary subunit, orange). (For interpretation of the references to colour in this figure legend, the reader is referred to the web version of this article.)

**Fig. 2:**
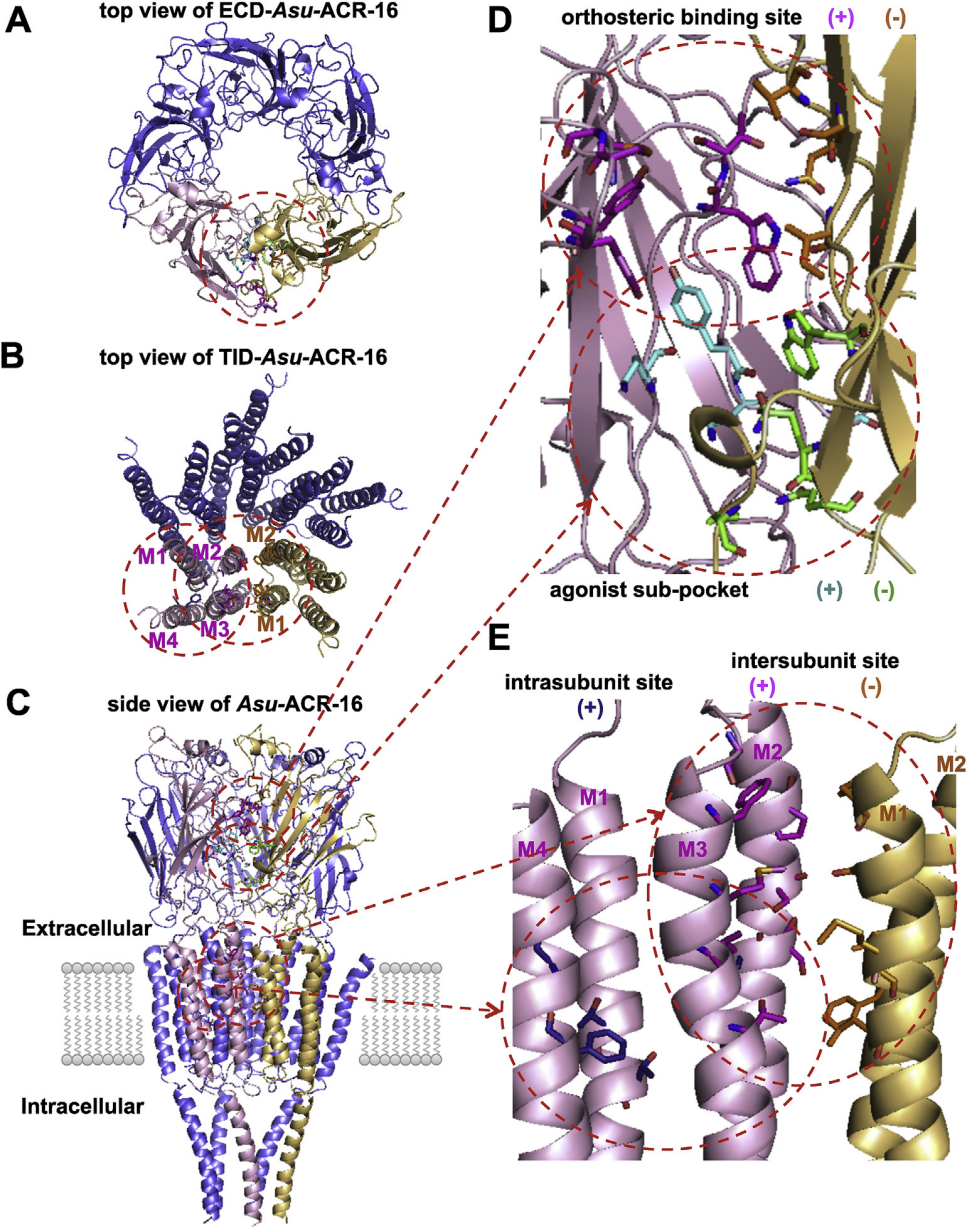
(A) Ribbon diagram of the antagonist-bound model of ECD-Asu-ACR-16 viewed from the synaptic cleft, showing the location of the orthosteric binding site and agonist sub-pocket. For clarity, only the front two subunits are highlighted (principal subunit, light pink; complementary subunit, yellow). The residues that contribute to the orthosteric binding site (principal side, pink; complementary side, orange) and the agonist sub-pocket (principal side, turquoise; complementary side, green) are represented by sticks and highlighted inside the red dotted circle. (B) Ribbon diagram of the antagonist-bound model of TID-Asu-ACR-16 viewed above the membrane, showing the location of two transmembrane allosteric binding sites. For clarity, only the front two subunits are highlighted (principal subunit, light pink; complementary subunit, yellow). The residues that contribute to the intersubunit site (principal side, pink; complementary side, orange) and intrasubunit site (principal side, purpleblue) are represented by sticks and highlighted inside the red dotted circle. (C) Ribbon diagram of the antagonist-bound model of full-length Asu-ACR-16 viewed parallel to the membrane plane, showing the location of the orthosteric binding site and the agonist sub-pocket in the extracellular domain, the intersubunit and intrasubunit binding sites in the transmembrane domain. For clarity, only the front two subunits are highlighted (principal subunit, light pink; complementary subunit, yellow). The residues that contribute to the ligand binding sites are represented by sticks (orthosteric site: (+), pink; (−), orange; agonist sub-pocket: (+), turquoise; (−), green; intersubunit transmembrane site: (+), pink; (−), orange; intrasubunit transmembrane site: purpleblue) and highlighted inside the red dotted circle. (D) Detailed view of the orthosteric binding site and agonist sub-pocket in the antagonist-bound model of ECD-Asu-ACR-16. The principal subunit is colored light pink, whereas the complementary subunit is colored yellow. The residues that contribute to the orthosteric binding site (principal side, pink; complementary side, orange) and the agonist sub-pocket (principal side, turquoise; complementary side, green) are represented by sticks and highlighted inside the red dotted circle. Carbon is in either turquoise or green. Nitrogen is in blue. Oxygen is in red. (E) Detailed view of the transmembrane allosteric binding sites in the antagonist-bound model of TID-Asu-ACR-16. The principal subunit is colored light pink, whereas the complementary subunit is colored yellow. The residues that contribute to intersubunit site (principal side, pink; complementary side, orange) and intrasubunit site (principal side, purpleblue) are represented by sticks and highlighted inside the red dotted circle. Carbon is in either pink or orange or purpleblue. Nitrogen is in blue. Oxygen is in red. Sulfur is in yellow. (For interpretation of the references to colour in this figure legend, the reader is referred to the web version of this article.)

The amino acid sequence of Asu-ACR-16 (Fig. 1) was obtained from the UniProtKB/SwissProt database with the accession number F1KYJ9 (Wang et al., 2011). Structural templates were identified by using BLASTP on NCBI network service (Altschul et al., 1997) and PSI-BLAST on the ProtMod server (Rychlewski et al., 2000) by searching in the Protein Data Bank (Berman et al., 2000). Three crystal structures of human α7 nAChR chimeras with different co–crystal ligands in orthosteric binding site were used: epibatidine bound (PDB code: 3SQ6; Li et al., 2011), no ligand (PDB code: 3SQ9; Li et al., 2011), and α-bungarotoxin bound (PDB code: 4HQP; Huang et al., 2013). These structures were selected as the templates for three different bound-forms of the ECD-Asu-ACR-16. The three models were: the agonist-bound form ECD-Asu-ACR-16; the apo form ECD-Asu-ACR-16 and; the antagonist-bound form ECD-Asu-ACR-16 (Fig. 2A).

We modeled the transmembrane and intracellular domains of Asu-ACR-16 (TID-Asu-ACR-16, Fig. 2B) because of the presence of an intrasubunit allosteric binding site that is found in α7 nAChR and an intersubunit allosteric binding site that is demonstrated in a Cys-loop receptor, GluCl crystal structure in complex with ivermectin (Young et al., 2008, Bertrand et al., 2008, Hibbs and Gouaux, 2011). Ivermectin is a known allosteric modulator of α7 nAChRs (Krause et al., 1998). The *T. marmorata* nAChR (PDB code: 2BG9 chain A; Unwin, 2005) is the only pentameric nAChR structure with the transmembrane domains and partial intracellular domains determined. Therefore, the transmembrane and intracellular domains of *T. marmorata* nAChR (TID-Tma-nAChR) were selected as the template for our TID-Asu-ACR-16 model.

The sequence of the ECD-Asu-ACR-16 and the human α7 nAChR chimera (SwissProt ID: P36544; Peng et al., 1994) were aligned using CLUSTALW multiple alignment (Thompson et al., 1994). The sequence of the TID-Asu-ACR-16 and TID-Tma-nAChR (SwissProt ID: P02711; Devillers-Thiery et al., 1983, Devillers-Thiery et al., 1984) were aligned using CLUSTALW.

### 2.2 Homology modeling of Asu-ACR-16

We used Modeller (Eswar et al., 2007) to build a three-dimensional model of ECD-Asu-ACR-16 and used JACKAL (http://wiki.c2b2.columbia.edu/honiglab_public/index.php/Software:Jackal) to build the model of TID-Asu-ACR-16 for each of the five subunits. These five subunits were then assembled to generate the pentamer using COOT software (Emsley and Cowtan, 2004). The model geometry was first refined manually, and then optimized by PHENIX software (Adams et al., 2010). Each of the TID-Asu-ACR-16 subunits were then merged into the ECD-Asu-ACR-16 model by using COOT to edit and alter the C_α_ coordinates of residues around the outer membrane regions. The final optimized pentameric model was then visualized using the program PyMol (The PyMOL Molecular Graphics System, Version 1.7.4, Schrödinger, and LLC., Figs. 2C & S1).

### 2.3 Structure-based virtual screening

Smiles strings of ligands were downloaded from the lead-like subset of commercially available compounds in the ZINC Database (Irwin et al., 2012) and were converted initially to PDB formats using the PHENIX-eLBOW program (Moriarty et al., 2009). The ligand and receptor input files were then prepared in PDBQT format for AutoDock Vina by using the AutoDock Tools package (Morris et al., 2009). For initial screening, a docking area was defined visually around the orthosteric binding site of ECD-Asu-ACR-16 (Fig. 2D S2A & S2B) by a grid box of 40 Å × 40 Å × 40 Å using 0.375 Å grid point spacing in AutoGrid. The conformations of ligands in the binding sites of the receptor were searched with GALS (Genetic Algorithm with Local Search; Morris et al., 1998). The binding free energies between the ligands and receptor were calculated by the combination of the knowledge-based and empirical scoring function in AutoDock Vina (Trott and Olson, 2010). The best nine binding modes of the ligand based on the binding affinities on the three bound-forms of ECD-Asu-ACR-16 models were implemented by AutoDock 20 runs for each ligand. Each docked ligand was then ranked by its highest binding affinity to the orthosteric binding site of the apo, agonist-bound, or antagonist-bound model. From the 60,000 screened molecules, we selected the top 9 ligands (0.015%) with the highest predicted affinities that had appropriate binding modes within the ligand-binding pockets for further study. We rejected those compounds without a cationic nitrogen in their structure and that were known to be: acutely toxic, or carcinogenic, or respiratory depressants, caused dermatitis or conjunctivitis or to be significant environmental hazards as recorded on the compound Safety Data Sheets available from Sigma Aldrich (http://www.sigmaaldrich.com/safety-center.html).

**Fig. 3:**
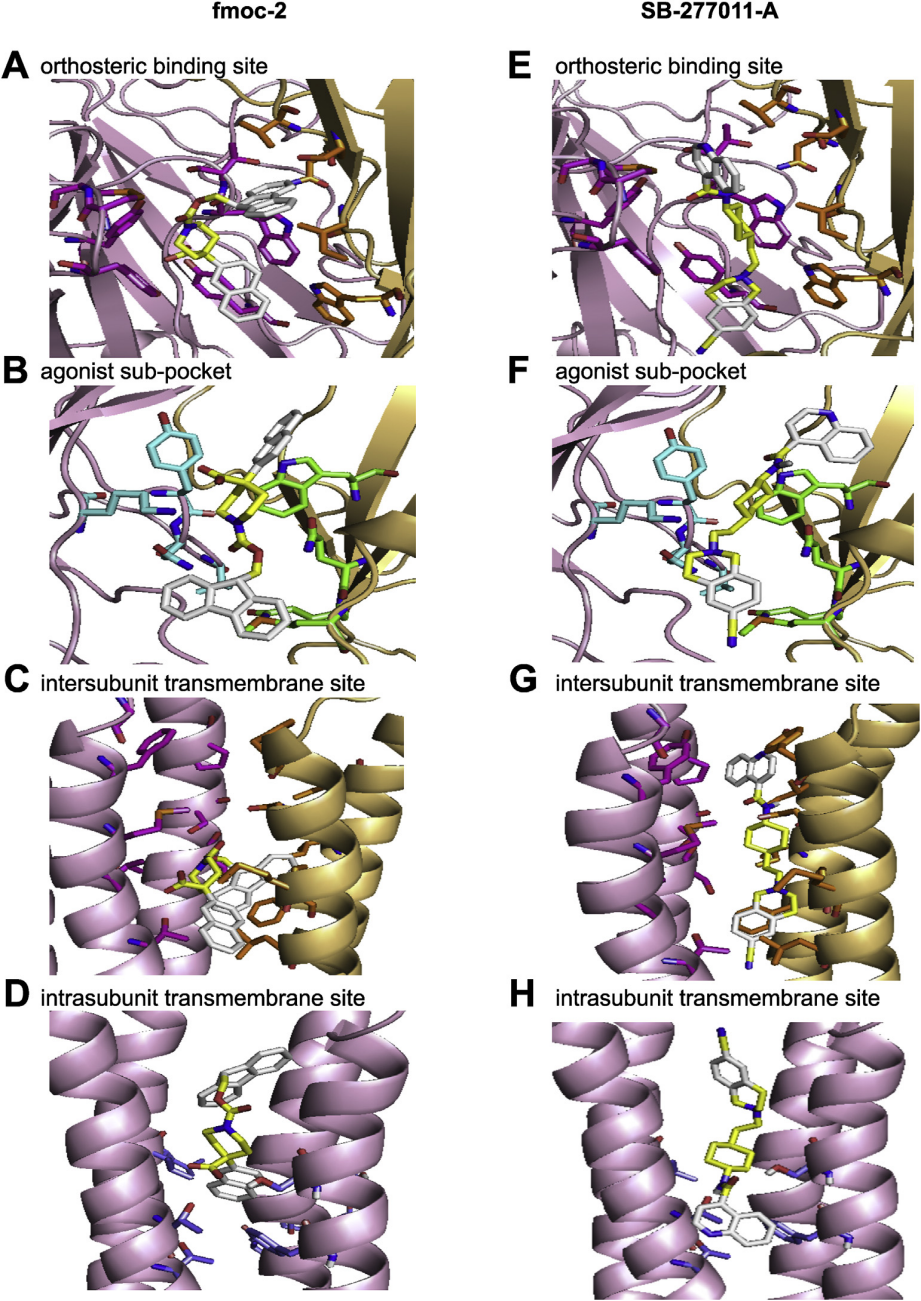

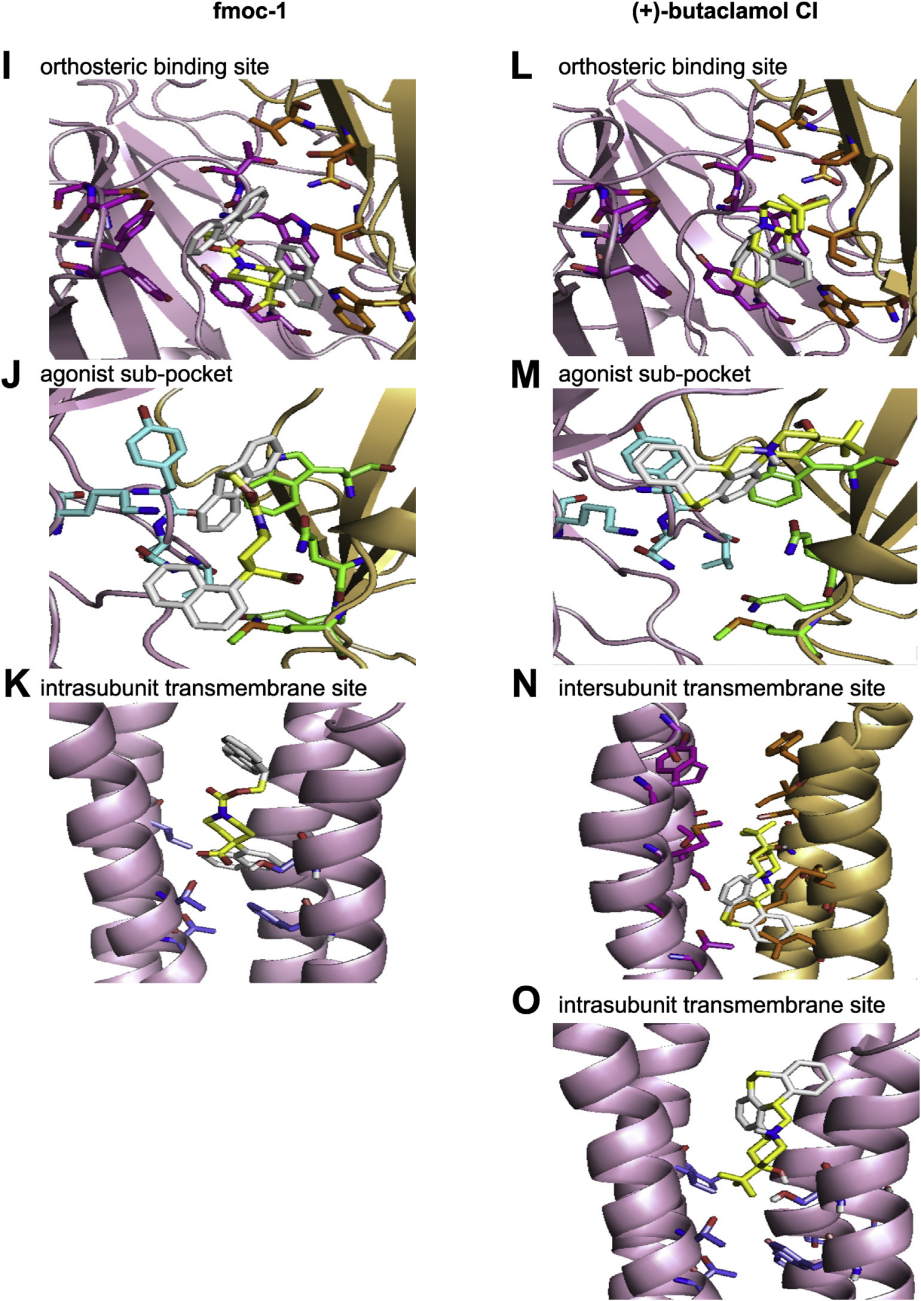
Binding modes of four virtual screening hits in the orthosteric binding site, the agonist sub-pocket, the intersubunit and intrasubunit transmembrane allosteric binding pockets of the antagonist-bound model of Asu-ACR-16: (A), (B), (C), (D) fomc-2; (E), (F), (G), (H) SB-277011-A; (I), (J), (K) fomc-1; (L), (M), (N), (O) (+)-butaclamol Cl. Hits docked into the binding pockets are represented by sticks (carbon in yellow; ring in white; nitrogen in blue; oxygen in red). (A), (E), (I) and (L) show the four hits bound in the orthosteric binding site of the antagonist-bound model of ECD-Asu-ACR-16. The front two subunits are highlighted (principal subunit, light pink; complementary subunit, yellow). The residues in the orthosteric binding site are labeled (principal side, pink; complementary side, orange) to show the location of the orthosteric binding site. (B), (F), (J) and (M) show the four hits bound in the agonist sub-pocket of the antagonist-bound model of ECD-Asu-ACR-16. The front two subunits are highlighted. The residues in the agonist sub-pocket are labeled (principal side, turquoise; complementary side, green) to show the location of the agonist sub-pocket. (C), (G) and (N) show the four hits bound in the intersubunit transmembrane site of the antagonist-bound model of TID-Asu-ACR-16. The front two subunits are highlighted. The residues in the intersubunit transmembrane site are labeled (principal side, pink; complementary side, orange) to show the location of the intersubunit transmembrane binding site. (D), (H), (K) and (O) show the four hits bound in the intrasubunit transmembrane site of the antagonist-bound model of TID-Asu-ACR-16. The residues in the intrasubunit transmembrane site are labeled (purpleblue) to show the location of the intersubunit transmembrane binding site. (For interpretation of the references to colour in this figure legend, the reader is referred to the web version of this article.)

The four virtual screen hits (Table 1) out of the top 9 selected ligands (44%) were also specifically docked into five allosteric binding pockets: the agonist sub-pocket (Fig. S2C & S2D); the vestibule pocket (Fig. S2E & S2F); the top pocket (Fig. S2G & S2H); the intersubunit and; the intrasubunit transmembrane sites (Fig. 2E S2I S2J). The docking area was defined visually around each allosteric binding pockets of Asu-ACR-16 by a grid box of 40 Å × 40 Å × 40 Å using 0.375 Å grid point spacing in AutoGrid. The docking was performed by AutoDock Vina (Fig. 3).

### 2.4 In vitro synthesis of cRNA and microinjection into *Xenopus laevis* oocytes

We used TRIzol (Invitrogen™) to extract the total RNA samples from a 1 cm muscle flap and dissected the whole pharynx of *A. suum*. The first-strand of cDNA was synthesized with oligo RACER primer, Random Hexamer and superscript III reverse transcriptase (Invitrogen, Carlsbad, CA, USA) from total RNA in the muscle and pharynx by reverse transcription polymerase chain reaction (RT-PCR). Full-length Asu-acr-16 cDNA was amplified with the forward primer TTGATGTAGTGGCGTCGTGT, ATCACGCATTACGGTTGATG and the reverse primer GCATTGATGTTCCCTCACCT, ATTAGCGTCCCAAGTGGTTG (Boulin et al., 2011). The XhoI and ApaI restriction enzymes were used to digest the amplified product, which was then cloned into pTB207 expression vector (Boulin et al., 2008) and linearized by NheI. We used the mMessage mMachine T7 kit (Ambion) to in vitro transcribe the linearized cDNA to cRNA, which was then precipitated with lithium chloride, re-suspended in RNase-free water, aliquoted and stored at −80 °C.

The ancillary protein RIC-3 is required for the expression of ACR-16 in Xenopus oocytes (Halevi et al., 2003). A 50 nL cRNA mixture was prepared with 25 ng Asu-acr-16 cRNA, 5 ng Asu-ric-3 cRNA (SwissProt ID: F1L1D9; Wang et al., 2011) dissolved in RNAse-free water. The nanoject II microinjector (Drummond Scientific, PA, USA) was used to injected the cRNA mixture into the animal pole of the de-folliculated *X. laevis* ​oocyte (Ecocyte Bioscience, Austin, TX, USA).

The injected oocytes were separated into 96-well culture plates and incubated in the incubation solution (pH 7.5), which is composed of 100 mM NaCl, 2 mM KCl, 1.8 mM CaCl_2_·2H_2_O, 1 mM MgCl_2_·6H_2_O, 5 mM HEPES, 2.5 mM Na pyruvate, 100 U/mL penicillin, 100 μg/mL streptomycin and changed daily. The injected oocytes were stored at 19 °C for 4–8 days to allow the receptor to be expressed.

### 2.5 Two-electrode voltage-clamp oocyte recording

We used two-electrode voltage-clamp electrophysiology to record the inward current generated by the activated Asu-ACR-16 receptors expressed in *X. laevis* oocytes. 100 μM BAPTA-AM (final concentration) was added into the oocyte incubation solution 4 h prior to recording, to prevent the current produced by the endogenous calcium-activated chloride channels during recording. An Axoclamp 2B amplifier (Molecular Devices, CA, USA) was used for recording and oocytes were held at −60 mV. A PC computer with software Clampex 9.2 (Molecular Devices, CA, USA) was used to acquire the recording data. The microelectrodes used to measure current in oocytes were pulled on a Flaming/Brown horizontal electrode puller (Model P-97, Sutter Instruments), filled with 3M KCl and had resistances of 20–30 MΩ. The microelectrode tips were broken back carefully with Kimwipes (Wilmington, NC, USA) to reduce the resistance to 2–5 MΩ. The recording solution was: 100 mM NaCl, 2.5 mM KCl, 1 mM CaCl_2_·2H_2_O and 5 mM HEPES, pH 7.3 (Buxton et al., 2014). Oocytes were placed into a tiny groove of the narrow oocyte recording chamber. The Digidata 1322A (Molecular Devices, CA, USA) was used to control the switches that controlled the perfusion of the chamber at a speed of 4–6 ml/min.

100 μM acetylcholine was applied initially for 10 s as a control to check the viability of the oocytes and Asu-ACR-16 expression for all the recordings. Recording solution was then used to wash out the drug from the oocytes for 2–3 min before next application of drug perfusion.

### 2.6 Drugs

Table 1 lists the compounds used, their chemical properties and structures. Fmoc-4-(naphthalen-2-yl)-piperidine-4-carboxylic acid (fmoc-2), SB-277011-A hydrochloride hydrate (SB-277011-A), fmoc-4-(naphthalen-1-yl)-piperidine-4-carboxylic acid (fmoc-1) and (+)-butaclamol hydrochloride ((+)-butaclamol Cl), acetylcholine chloride (ach), methyllycaconitine citrate salt (mla) were purchased from Sigma–Aldrich (St Louis, MO, USA). Levamisole hydrochloride (levamisole) was purchased from MP Biomedicals (Santa Ana, CA, USA). With the exception of ach and mla which were dissolved in the recording solution, the rest of chemicals were dissolved in dimethyl sulfoxide (DMSO) to make stock solutions. Stock solutions of 100 mM were prepared, except for SB-277011-A where a stock solution of 10 mM was prepared due to the solubility; stock solutions were frozen until required. Working solutions were then prepared by dilution on the day of the experiment.

### 2.7 Pharmacological characterization of molecules selected by virtual screening

To characterize the four hits (Table 1) selected by our virtual screening, each drug was applied for 10 s to the oocytes expressing Asu-ACR-16 to test if the drugs were agonists. They were then tested as antagonists against ach.

**Fig. 4:**
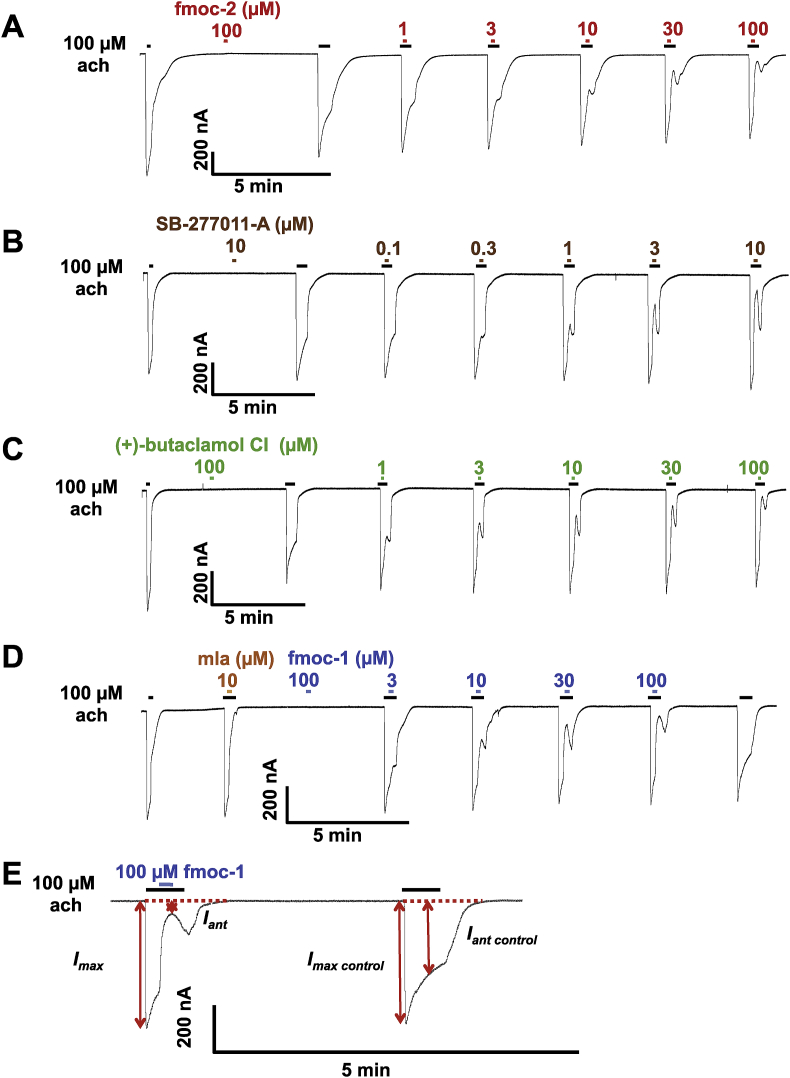
Effects of four virtual screening hits on Asu-ACR-16 mediated ach responses. Sample traces for: (A) fmoc-2, (B) SB-277011, (C) (+)-butaclamol Cl, (D) fmoc-1 concentration-inhibition relationships on Asu-ACR-16. Mla in (D), which stands for methyllycaconitine citrate salt, was used as an antagonist control of Asu-ACR-16. All four hits did not induce the current response by themselves, while produced the concentration-depended inhibition of ach current response. (E) is the magnified figure of part of (D) as an example to show the four parameters needed to measure the inhibition percentage. I_max control_ was the peak current of the control 30 s application of 100 μM ach. I_max_ was the peak current of the 100 μM ach that preceded the 10 s co-application of ach and antagonist. I_ant_ was the minimal current during the co-application of 100 μM ach and antagonist. I_ant control_ was the current at the same time point from the beginning of the 30 s application as I_ant_ during the control 30 s application of 100 μM ach.

To characterize the antagonistic properties of the four hits, the following protocol was used: a) 10 s of 100 μM ach alone; b) then 10 s of 100 μM ach + hit and then; c) 10 s of 100 μM ach alone. This test procedure was repeated with increasing concentrations of the four hits (Fig. 4A–D), to determine the inhibitory dose–response relationships and IC_50_ by fitting Hill equations to the inhibitory dose–response curves using GraphPad Prism 5.0 (Graphpad Software Inc., CA, USA). As a further study of the antagonism, each of the four hits was applied before and during 10 s test applications of increasing concentrations of ach (Fig. S4).

### 2.8 Data analysis

The data from electrophysiological recordings were analyzed using Clampfit 9.2 (Molecular Devices, CA, and USA) and GraphPad Prism 5.0 (Graphpad Software Inc., CA, USA). In all recordings, the peak currents in response to applied drugs were measured, which were later normalized to the control 100 μM ach response, and expressed as mean ± S.E.M. The mean % inhibition of currents elicited by 100 μM ach ± S.E.M. was used to determine the inhibition percentage, which was quantified using the following equation:

$$\mathrm{Inhibition}\left(\%\right)=\left(1-\;\frac{I_{\mathrm{ant}}}{\frac{I_{\mathrm{ant control}}}{I_{\mathrm{maxcontrol}}}\;\times \;I_{\max}}\;\right)\times 100\text{\%}$$

where I_max control_ was the peak current of the control 30 s application of 100 μM ach, I_max_ was the peak current of the 100 μM ach that preceded the 10 s co-application of ach and antagonist. I_ant_ was the minimal current during the co-application of 100 μM ach and antagonist. I_ant control_ was the current at the same point from the beginning of the 30 s application as I_ant_ during the control 30 s application of 100 μM ach (Fig. 4E). Concentration-response relationships or concentration-inhibition (%) relationships were analyzed by fitting data points into the Hill equation, with at least four replicates of each experiment set.

### 2.9 Drug treatment of *C. elegans*

The wild-type *C. elegans* strain N2 were obtained from the Caenorhabditis Genetics Center (University of Minnesota, MN, USA). We grew *C. elegans* at 20 °C on nematode growth media (NGM, 3 g/l NaCl, 17 g/l agar, 2.5 g/l peptone, 1 mM CaCl_2_, 5 mg/l cholesterol, 1 mM MgSO_4_, 25 mM KPO_4_ buffer) agar plates, seeded with *Escherichia. coli* OP50 lawn under standard conditions (Brenner, 1974). Ten larvae at L4 stage with active thrashing movement (defined as “normal”) were transferred from NGM plates into M9 buffer (3 g/l KH_2_PO_4_, 6 g/l Na_2_HPO_4_, 5 g/l NaCl, 1 mM MgSO_4_) in 24-wall plates for each treatment. We counted the number of worms with normal motility in M9 buffer with diluted drugs from the stock solutions (≤1% DMSO) at 0, 5, 10, 15 and 20 min. Five replicates were applied for each treatment. Motility between negative control (1% DMSO, final concentration) and drug treated worms were compared at each time point using student t-test.

## 3 Results

### 3.1 Sequence alignment of Asu-ACR-16 and template homologue proteins

The full-length protein sequence of Asu-ACR-16 (504 residues) was retrieved from the SwissProt database, of which the ECD-Asu-ACR-16 accounts for 234 residues. The first 25 resides of Asu-ACR-16 were excluded from alignment with the full length human nAChR α7 chimera (204 residues) because of the shorter length of the template protein sequence. The human α7 nAChR chimera shows 37.6% sequence identity and 72.9% sequence similarity with the ECD-Asu-ACR-16, based on the alignment generated by CLUSTALW (Fig. 1A, job ID: 65782ad6ad6d). The TID-Tma-nAChR subunit A shows 22.0% sequence identity and 45.4% sequence similarity with TID-Asu-ACR-16, aligned by CLUSTALW (Fig. 1B, job ID: 644888f4f30e). The residues involved in the putative orthosteric and the allosteric binding sites are highlighted in amino acids sequence of Asu-ACR-16.

### 3.2 Models of the Asu-ACR-16 pentamer

The model of the antagonist-bound form of the ECD-Asu-ACR-16 subunit starts from an N-terminal α helix followed by seven β strands that comprise an immunoglobulin fold. Loop A (Val114 – Ala122), loop B (Lys169 – Lys179), loop C (Phe213 – Pro220) from the principal subunit, and loop D (Ala78 – Ala83), loop E (Ile143 – Pro144), loop F (Gly185 – Met204) from the complementary subunit are involved in forming the orthosteric binding site. A disulphide bond between Cys152 and Cys166 contributes to the characteristic component of Cys-loop receptors. The C-terminal continues into the transmembrane domain (Fig. S1A).

The transmembrane domains of the Asu-ACR-16 model are made of four α-helices (M1, M2, M3 and M4). M1 links to the β7 sheet of the extracellular domain and extends down into the membrane and is followed by the M2 and the M3 helixes as the membrane-spanning portions. The MA cytoplasmic loop (helix) connects between M3 and M4. The region between M3 and MA is not modeled due to the poorly defined intracellular domain of the template structure. The C-terminal follows the M4 helix and faces toward the extracellular surface (Fig. S1B).

The pentameric model of Asu-ACR-16 has a five-fold symmetric around the channel pore. The average pairwise Root Mean Square Deviation (RMSD) fit of the C_α_ coordinates of the antagonist-bound ECD-Asu-ACR-16 pentameric model and human α7 nAChR chimera pentamer (PDB code: 4HQP) was 0.9 Å, which indicates a strong structural conservation between the model and the template structures (Fig. S1C). The C_α_-RMSD between the TID-Asu-ACR-16 pentamer and the TID-Tma-AChR pentamer was 1.5 Å, which shows the TID fit is still good but not as good as the ECD fit. The membrane-spanning domains are arranged symmetrically. The M2 helix lines the channel pore, while M1, M3 and M4 do not contribute to the channel pore and are arranged peripherally (Fig. S1D).

Since no binding site data of Asu-ACR-16 is available to date, we used the published orthosteric binding site and allosteric binding sites in nAChRs or other Cys-loop receptors to predict the putative binding sites in Asu-ACR-16 (Galzi et al., 1991, Arias, 2000, Young et al., 2008, Hibbs and Gouaux, 2011, Spurny et al., 2015). The orthosteric binding site is at the interface between the principal site and the complementary site in two adjacent subunits of the ECD-Asu-ACR-16 pentamer (Fig. 2 S2A & S2B). The principal subunit (+) has vicinal cysteines (Cys216, Cys217) that contributes to the loop C of the binding site. The complementary subunit (−) does not use vicinal cysteines as part of the binding pocket and the residues are more variable when nAChRs are compared. The agonist sub-pocket, which we argue is a less significant allosteric binding site in ECD-Asu-ACR-16, is located right below the orthosteric binding site in the extracellular domain (Fig. 2 S2C & S2D). The vestibule pocket (Fig. S2E & S2F) and the top pocket (Fig. S2G & S2H) were not high affinity binding sites for the ligands and are not discussed further in this manuscript. The intersubunit allosteric binding sites in TID-Asu-ACR-16 are at the interface region between M2(+), M3(+), M1(−) and M2(−) (Fig. 2 S2I & S2J). The intrasubunit allosteric binding sites are at the center of the four transmembrane helixes (M1, M2, M3 and M4) in each of the five subunits.

### 3.3 Binding properties of virtual screening hits

We carried out virtual screening of the ZINC ligand-database by using the three different bound forms of the ECD-Asu-ACR-16 models. Four molecules were selected as hits based on their high binding affinities and appropriate binding modes within the ligand-binding sites. The 9-fluorenylmethoxycarbonyl group (FMOC) was observed in twelve out of top forty hits ranked by binding affinities and exists in the two out of four hits, which suggests that FMOC could be necessary for the ligand recognition by the receptor. The FMOC group has a low predicted bioavailability due to the biphenyl scaffold, which limits aqueous solubility and may affect distribution to the *A. suum* parasite. Table 1 lists the physicochemical characteristics of four hits. They have relatively high molecular weights and are more hydrophobic compared to known Asu-ACR-16 agonists. However, they do follow the Lipinski's rule of five, which suggests that these molecules may be orally actively (Lipinski et al., 2001, Lipinski, 2004).

The atomic structure predicts the partition-coefficients (XlogP) of the four hits to be between 4.27 and 6.04 (Table 1). The XlogPs suggest that the four hits are 10,000–1,000,000 times more concentrated in the lipophilic phase of the lipid bilayer than the aqueous phase of the extracellular domain (Cheng et al., 2007). The four hydrophobic hits are, therefore, more likely to bind into the transmembrane allosteric binding pockets rather than to the extracellular ligand binding sites. The four hits which bind in the transmembrane allosteric binding pockets are therefore predicted to be allosteric modulators of the Asu-ACR-16 receptor that alter the activity of the agonists or competitive antagonists that bind to orthosteric binding site. SB-277011-A is known to be a potent and selective dopamine D_3_ receptor antagonist with high oral availability (Stemp et al., 2000). (+)-butaclamol Cl is a non-selective dopamine receptor antagonist and a potent antipsychotic agent (Chrzanowski et al., 1985). No paper reporting on the activities of fmoc-2 and fmoc-1 has been published to date.

The four hits (Table 1) were tested for docking into the orthosteric binding sites of the three forms of ECD-Asu-ACR-16 models and the five allosteric binding pockets in the antagonist-bound form of full-length Asu-ACR-16 models. All four hits bound to the orthosteric binding sites of three ECD-Asu-ACR-16 models, but only bound to the three allosteric binding sites out of five: the intersubunit and intrasubunit transmembrane pockets and the agonist sub-pocket (Fig. 3) with high binding affinities.

In the intersubunit transmembrane site of TID-Asu-ACR-16 model, M243 (M1, (−)), L247 (M1, (−)) make hydrophobic interactions with naphthalene of fmoc-2. T312 (M3, (+)), S284 (M2, (+)) form hydrogen bonds with carboxylic acids of fmoc-2. F279 (M2, (−)), I282 (M2, (−)) and make hydrophobic contacts with fluorene of fmoc-2. F279 (M2, (−)), P244 (M1, (−)) make hydrophobic interactions with tetrahydroisoquinoline of SB-277011-A. N240 (M1, (−)) forms a hydrogen bond with carboxamide of SB-277011-A. P288 (M2, (+)) has hydrophobic interactions with quinoline of SB-277011-A. L247 (M1, (−)), F279 (M2, (−)) and makes hydrophobic contacts with dibenzocycloheptene of (+)-butaclamol Cl.

Ach, the natural agonist of Asu-ACR-16 was docked into the ligand binding sites of three forms of Asu-ACR-16 models for comparison. As expected, ach bound to the orthosteric binding site of the agonist-bound Asu-ACR-16 with an affinity (−4.3 kcal/mol), which was higher than the affinities at the other binding sites. The binding pose of ach docked in the orthosteric binding site of the agonist-bound Asu-ACR-16 model was in agreement with the binding pose of ach in the *L. stagnalis* AChBP cocrystal structure (PDB code: 3WIP; Olsen et al., 2014b). The quaternary ammonium of ach faces the basal side of the binding cavity and make cation-π interaction with five aromatic residues from the Asu-ACR-16 ((+): Y89, W143, Y185, Y192; (−): W53), while the carbonyl oxygen of ach faces toward the apical side of the binding cavity. The binding affinities of the selected four compounds were higher than −8.0 kcal/mol in the three different bound forms of Asu-ACR-16, while the binding affinities of ach were lower than −4.5 kcal/mol in three states of Asu-ACR-16 (Table 2).

### 3.4 Pharmacological properties of virtual screening hits

**Fig. 5:**
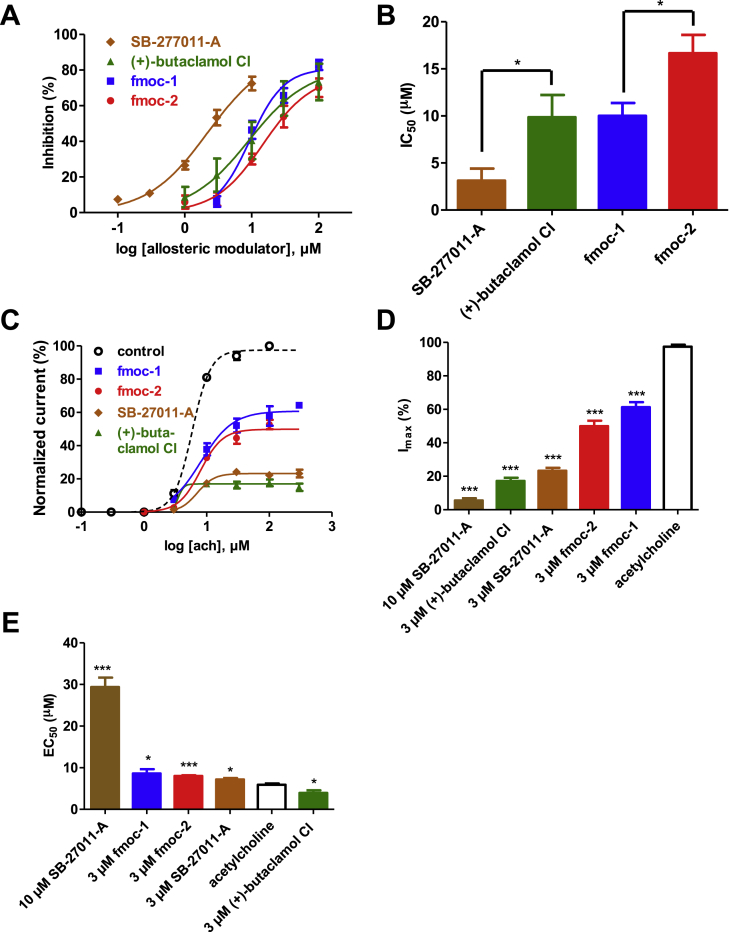
(A) Effects of four virtual screening hits on Asu-ACR-16 mediated ach responses. Fmoc-2, fmoc-1, (+)-butaclamol Cl and SB-277011-A concentration-inhibition curves for Asu-ACR-16. Results were expressed as mean % inhibition of currents elicited by 100 μM ach ± S.E.M. (B) Bar chart representing the IC50 (mean ± S.E.M, μM) of each plots in (A). The rank order series of inhibition based on IC50 for four hits is: SB-277011-A (3.12 ± 1.29 μM, n = 4) < (+)-butaclamol Cl (9.85 ± 2.37 μM, n = 4) ≈ fmoc-1 (10.00 ± 1.38 μM, n = 4) < fmoc-2 (16.67 ± 1.95 μM, n = 4). * represents p < 0.05 (unpaired t-test). (C) Ach concentration-response plots for Asu-ACR-16 in the absence of hits as a control (ach) and in the continual presence of four hits identified in (A). Ach concentration-response curves for Asu-ACR-16 in the presence of 3 μM of four hits: fmoc-1, fmoc-2, SB-27011-A and (+)-butaclamol Cl. (D) Bar chart (mean ± S.E.M, %) representing the reduced maximum current response of ach concentration-response curves in (C). The series of reduced maximum response of each hits compared to that of ach by unpaired t-test is: 10 μM SB-27011-A (5.51 ± 1.38%, n = 4), 3 μM (+)-butaclamol Cl (17.22 ± 1.94%, n = 4), 3 μM SB-27011-A (23.25 ± 1.80%, n = 5), 3 μM fmoc-2 (49.92 ± 3.27%, n = 4), 3 μM fmoc-1 (61.25 ± 3.08%, n = 4) and ach (97.45 ± 1.19%, n = 4). * represents p < 0.05, ** represents p < 0.01, *** represents p < 0.001. All four hits significantly inhibited the maximum current response induced by ach. (E) Bar chart (mean ± S.E.M, μM) displaying the EC50 of ach concentration-response curves in (C). The series of variable EC50 of each hits compared to that of ach by unpaired t-test is: 10 μM SB-27011-A (29.40 ± 2.27 μM, n = 4), 3 μM fmoc-1 (8.62 ± 1.04 μM, n = 4), 3 μM fmoc-2 (8.01 ± 0.18 μM, n = 4), 3 μM SB-27011-A (7.17 ± 0.33 μM, n = 5), ach (5.92 ± 0.29 μM, n = 4) and 3 μM (+)-butaclamol Cl (3.94 ± 0.66 μM, n = 4). The EC50 of all four hits obviously shift away from the control when applied.

We tested the effects of the putative allosteric modulators on Asu-ACR-16 receptors expressed in Xenopus oocyte using two-electrode voltage clamp to observe the currents that flow through Asu-ACR-16 receptors. Representative traces showing the inhibitory dose–response relationships are shown in Fig. 4. Their IC_50_ (Fig. 5A and B) and maximum inhibition (Fig. S3) were determined as described in the methods (Table 3). The most potent antagonist among them was SB-277011-A, which had an IC_50_ of 3.12 ± 1.29 μM and maximum inhibition effect of 96.07 ± 10.66% (n = 4).

The ach concentration-response plots in the presence of 3 μM of each putative allosteric modulator (Fig. S4 & Fig. 5C), show the reduced maximum current responses with little shift in EC_50_ of ach (Fig. 5Dand E & Table 4), and that the hits were non-competitive antagonists and negative allosteric modulators.

At 10 μM, SB-277011-A, showed evidence of a mixed competitive and non-competitive antagonism (Fig. S5), characterized by a reduced maximum current response and a right-shift in the EC_50_ of ach (Fig. 5D and E). Thus, 10 μM SB-277011-A appears to act at more than one binding site which may include the orthosteric binding sites and additional allosteric binding sites.

### 3.5 SB-277011-A reversibly inhibits locomotion in *C. elegans*

We tested the effects of each allosteric modulator on the locomotion of *C. elegans* L4 larvae. The number of normal worms with thrashing-like movement dropped by 60% in 5 min after exposed to 30 μM SB-277011-A (p < 0.01, n = 5, t-test). Paralysis-like movement was observed in the rest of the worms. Interestingly, the number of worms with motility that appeared normal recovered to 50% (p < 0.05, n = 5, t-test) in 10 min, 85% in 15 min (p > 0.05, n = 5, t-test) and returned to near negative control values after 20 min (Fig. S6). The recovery may relate to the desensitization properties of the ACR-16 receptor. The reversible inhibition of motility in worms was also observed in 100 μM (+)-butaclamol Cl, but no significant difference between the number of normal treated worms and negative control was observed at any time point. No visual effects of 100 μM fmoc-2 or 100 μM fmoc-1 were found on the locomotion of worms.

## 4 Discussion

### 4.1 Asu-ACR-16 models

We have built up three-dimensional models of full-length structures of Asu-ACR-16 at the atomic level for the first time. We used homology modeling based on X-ray crystal structures of human α7 nAChR chimeras and the electron microscopic structure of the *T. marmorata* nAChR as templates for different domains. The quality of our homology models are dependent on the sequence identity of the templates (human α7 nAChR chimeras and *T. marmorata* nAChR) and the target sequence (Asu-ACR-16) and the resolutions of template structures (Hillisch et al., 2004, Cavasotto and Phatak, 2009). Our three ECD-Asu-ACR-16 models are likely to be reliable for virtual screening because they have high sequence identities (37.6% identity and 72.9% similarity) with high resolution (<4 Å) templates. More errors might be expected in the TID-Asu-ACR-16 model, because of the missing loop between M3 and MA in the template structure which reduces sequence identity with the target protein. The missing loop does not include an allosteric binding site, so we can assume that the TID-Tma-nAChR structure is similar to the TID-Asu-ACR-16 structure (Bertrand et al., 2008). The overall secondary structures of our models are also consistent with published nAChRs structures (Finer-Moore and Stroud, 1984, Miyazawa et al., 2003, Unwin, 2005).

We developed the apo, the agonist-bound and the antagonist-bound models of the ECD-Asu-ACR-16 on the assumption that these three states of the Asu-ACR-16 receptor most closely represent the receptor conformations in the presence and absence of agonists or antagonists. To produce a realistic dynamic model would require more extensive work (Cavasotto and Orry, 2007, Spyrakis et al., 2011) and is beyond the scope of this study.

### 4.2 Virtual screening

Our structure-based virtual screening approach identified four novel and potent negative allosteric modulators of Asu-ACR-16, which were validated by our electrophysiological studies. The putative ligands were initially selected based on the virtual screening using the orthosteric binding site of the receptor. It was possible that these ligands could have been agonists or competitive antagonists that bind within the orthosteric binding site. In contrast, the pharmacological characterization of the four virtual screening hits shows that they behave as negative allosteric modulators and bind to allosteric sites. This outcome may be due to the hydrophobic properties of the four compounds that impedes their interactions with the orthosteric site in the extracellular domain of the receptor. The high lipid solubility of these compounds increases their concentration in the membrane lipid phase, in the region of the transmembrane allosteric sites.

The binding affinities calculated in the scoring function of AutoDock Vina software usually increase with the number of non-hydrogen atoms, which may be due to the neglect of desolvation in the scoring function (Shoichet et al., 1999, Kuntz et al., 1999, Park et al., 2006). This leads to a bias of virtual screening methods towards big molecules which are more hydrophobic, concentrated in the lipid bilayer, and less likely to interact with the binding sites in the extracellular domains (Hopkins et al., 2004). It is also pointed out that the simplified force fields used to estimate the binding free energies are unable to evaluate the conformational entropies and other contributions to the free energies (Cosconati et al., 2010). Thus, the success rate of identifying bioactive hits (44%) would be enhanced if we are able to include these additional parameters into a scoring function for virtual screening. Another approach, which we did not follow here, to enhance the success rate of identifying bio-active hits, is to use the known agonists or antagonists as scaffolds. This would facilitate the identification of low molecular-weight and more hydrophilic agonists or antagonists, and allow further study of the quantitative structure-activity relationships (Sun, 2008).

### 4.3 Four negative allosteric modulators of Asu-ACR-16

We evaluated the potency of inhibition for the four negative allosteric modulators in our electrophysiology studies on *Xenopus* oocytes: SB-277011-A (IC_50_ 3.12 ± 1.29 μM) < (+)-butaclamol Cl (IC_50_ 9.85 ± 2.37 μM) ≈ fmoc-1 (IC_50_ 10.00 ± 1.38 μM) < fmoc-2 (IC_50_ 16.67 ± 1.95 μM). This rank of inhibition agrees with the level of effects of the four modulators in the motility of *C. elegans*. The most potent modulator SB-277011-A was shown to decrease the motility of *C. elegans* larvae for a duration of about 10 min, yet less effective on adult *C. elegans*. Desensitization of the ACR-16 or other nAChRs in *C*. *elegans* body muscle may be a reason for the reduced effects of SB-277011-A on worms (Hernando et al., 2012). Treating the acr-16-null mutant of *C. elegans* with SB-277011-A can help us to investigate the mode of action of SB-277011-A on *C. elegans* as genetic models to understand SB-277011-A action on the parasitic nematode *A. suum* (Ward, 2015).

### 4.4 Allosteric binding sites may offer a better opportunity for drugs that can discriminate between the parasite Asu-ACR-16 and mammalian host α7 nAChR

Asu-ACR-16 shows 42.5% sequence identity and 71.2% sequence similarity with the human α7 nAChR (SwissProt ID: P36544) based on the alignment generated by CLUSTALW (Fig. S7, CLUSTALW job ID: cfed4f821eaf). The residues constituting the orthosteric binding site (pink and orange arrows in Fig. S7) are highly conserved between Asu-ACR-16 and human α7 nAChR, which shows 66.7% identity and 100% similarity (Fig. S8). In contrast, the residues of the four allosteric binding sites have much greater differences (variance) between the nematode parasite and the equivalent sites on the α7 receptor (identities: 62.5%, 45.5%, 66.7%, 62.5% and 40.0% and; similarities: 87.5%, 81.8%, 83.3, 93.8% and 100%). The sequence divergence in the allosteric binding sites between Asu-ACR-16 and host human α7 nAChR indicates that drugs targeted at these sites may be more selective than drugs targeted at orthosteric binding sites. Virtual screening specifically targeting the allosteric binding sites is predicted to offer a better opportunity for development of drugs with much greater receptor subtype selectivity (Nussinov and Tsai, 2013, Iturriaga-Vasquez et al., 2015).

### 4.5 Conclusion

We have developed a structure-based in silico screening approach to search for the bioactive hits that target at a parasitic nematode receptor. This approach allowed us to identify four negative allosteric modulators that were validated using our electrophysiological studies. These four compounds may be useful leads for anthelminthic drug discovery. We point out however, that we have not yet made the structural models for the host human α7 nAChR or other receptors, which would help to distinguish compounds that are active only on the nematode receptors, thereby reducing potential toxicity. It would also be desirable to perform virtual screening for toxicity on a range of host receptors, some structures of which have already been determined and others need to be modeled.

## Statement of conflict of interest

None identified.

## Figures and Tables

**Table 1:** Physicochemical properties and chemical structures of four virtual screen hits. The four hits are: fmoc-4-(naphthalen-2-yl)-piperidine-4-carboxylic acid (fmoc-2), SB-277011-A hydrochloride hydrate (SB-277011-A), fmoc-4-(naphthalen-1-yl)-piperidine-4-carboxylic acid (fmoc-1), (+)-butaclamol hydrochloride ((+)-butaclamol Cl). The molecular mass (Mol. Mass), number of hydrogen bondonors, number of hydrogen bond acceptors, number of rotatable bonds and partition coefficient (xlogP) are listed for each hits.

ZINC ID	44122512	26574567	44122502	02008410
Hits	fmoc-2	SB-277011-A	fmoc-1	(+)-butaclamol Cl
Structure				
Mol. Mass	478	475	478	363
H-bondonors	0	1	0	2
H-bond acceptors	5	5	5	2
Rotatable bonds	5	5	5	1
XlogP	6.04	4.27	6.02	4.96

**Table 2:** Binding affinities (kcal/mol) of the four hits and ach in the orthosteric binding sites of the three different bound models of ECD-Asu-ACR-16 and three allosteric binding sites of the antagonist-bound model of full-length Asu-ACR-16.

Hits	Binding affinities (kcal/mol)					
Apo	Agonist-bound	Antagonist-bound		Transmembrane site	
Orthosteric binding site	Orthosteric binding site	Orthosteric binding site	Agonist sub-pocket	Inter-subunit	Intra-subunit
fmoc-2	−10.4	−13.0	−10.2	−8.5	−11.4	−10.8
SB-277011-A	−9.8	−12.3	−9.2	−9.5	−10.8	−9.7
fmoc-1	−10.6	−12.3	−10.3	−8.9	NA	−11.0
(+)-butaclamol Cl	−9.5	−11.8	−8.2	−9.2	−10.8	−9.0
ach	−4.2	−4.3	−3.9	−4.0	−3.7	NA

**Table 3:** Pharmacological profiles of the inhibitory effects of four hits on Asu-ACR-16 mediated ach responses. Results (mean ± S.E.M.) were expressed as IC_50_ (μM), Hill slope (n_H_), maximum inhibition (%) and the number of repeats (N) of each experiment.

Hits	IC_50_ (μM)	nH	Inhibitionmax (%)	N
fmoc-2	16.67 ± 1.95	1.22 ± 0.17	80.34 ± 10.32	4
SB-277011-A	3.12 ± 1.29	0.99 ± 0.11	96.07 ± 10.66	4
fmoc-1	10.00 ± 1.38	1.97 ± 0.37	82.49 ± 4.74	4
(+)-butaclamol Cl	9.85 ± 2.37	1.34 ± 0.29	79.53 ± 12.41	4

**Table 4:** Pharmacological profiles of EC_50_ shifts and maximum current reductions of Asu-ACR-16 mediated ach responses in the presence and absence of four hits. Results (mean ± S.E.M.) were expressed as EC_50_ (μM), Hill slope (nH) and maximum response (%) and the number of repeats (N) of each experiment.

Hits	EC_50_ (μM)	nH	Imax (%)	N
control	5.92 ± 0.29	3.05 ± 0.07	97.45 ± 1.19	4
3 μM fmoc-2	8.01 ± 0.18	2.54 ± 0.18	49.92 ± 3.27	5
1 μM SB-277011-A	9.85 ± 4.80	2.60 ± 0.71	75.96 ± 4.40	4
3 μM SB-277011-A	7.17 ± 0.33	3.37 ± 0.28	23.25 ± 1.80	5
10 μM SB-277011-A	29.40 ± 2.27	7.43 ± 3.30	5.51 ± 1.38	4
3 μM fmoc-1	8.62 ± 1.04	1.76 ± 0.21	61.25 ± 3.08	4
1 μM (+)-butaclamol Cl	4.54 ± 0.92	9.64 ± 4.14	30.34 ± 4.03	4
3 μM (+)-butaclamol Cl	3.94 ± 0.66	12.75 ± 3.63	17.22 ± 1.94	4

## Appendix A Supplementary data

Figure S1. (A) Ribbon representation of the ECD-Asu-ACR-16 monomer. Secondary elements are indicated. Loop A, B, C in the principal subunit and loop D, E, F in the complementary subunit which mainly contribute to the orthosteric binding site are labeled by dark green. In the complete structure, the C-terminal would enter the membrane at the bottom and link to M1 helix in the transmembrane domain. (B) Ribbon representation of the full-length Asu-ACR-16 monomer, as viewed parallel to the membrane plane. Four α helixes (M1, M2, M3 and M4) that contribute to transmembrane domain and one MA helix that makes up the intracellular domain are indicated. The functionally important M1-M2, M2-M3 and MA-M4 loops are labeled. (C) Superposition of antagonist-bound model of ECD-Asu-ACR-16 pentamer (purple blue) and template human α7 nAChR chimera (red; PDB code: 4HQP, ligands removed for clarity) viewed from the synaptic cleft. Five homomeric α-subunits are labeled. The interfaces between two vicinal α-subunits are marked by dotted lines. Five orthosteric binding sites are at each interface between the principal side (+) and the complementary side (−) from two vicinal subunits. (D) Superposition of TID-Asu-ACR-16 pentamer (purple blue) and template TID-Tma-AChR (red; PDB code: 2BG9) viewed above the membrane. Five homomeric α-subunits are labeled. The interfaces between two vicinal α-subunits are marked by dotted lines. Five allosteric binding sites are at each interface among the M2, M3 in the principal side (+) and the M1 in the complementary side (−) from two vicinal subunits.

Figure S2. (A) Detailed view of the orthosteric binding site in the antagonist-bound model of ECD-Asu-ACR-16. Principal subunit is colored light pink, whereas the complementary subunit is colored yellow. The residues that contribute to the orthosteric binding site are represented by sticks (principal side (+), pink; complementary side (−), orange) and highlighted inside the red dotted circle. Carbon is in either pink or orange. Nitrogen is in blue. Oxygen is in red. Sulfur is in yellow. (B) Detailed view of the residues involving in the orthosteric binding site in the antagonist-bound model of ECD-Asu-ACR-16. Functionally important amino acids which interact with the ligands bound in this region are labeled. (C) Detailed view of the agonist sub-pocket relative to the location of the orthosteric binding site in the antagonist-bound model of ECD-Asu-ACR-16. Principal subunit is colored light pink, whereas the complementary subunit is colored yellow. The residues that contribute to the agonist sub-pocket are represented by sticks (principal side (+), turquoise; complementary side (−), green) and highlighted inside the red dotted circle at the bottom. Carbon is in either turquoise or green. Nitrogen is in blue. Oxygen is in red. (D) Detailed view of the residues involving in the agonist sub-pocket in the antagonist-bound model of ECD-Asu-ACR-16. Functionally important amino acids which interact with the allosteric modulators bound in this region are labeled. (E) Detailed view of the vestibule pocket relative to the location of the orthosteric binding site in the antagonist-bound model of ECD-Asu-ACR-16. Principal subunit is colored light pink, whereas the complementary subunit is colored yellow. The residues that contribute to the vestibule pocket are represented by sticks (principal side (+), dark green; complementary side (−), gold) and highlighted inside the red dotted circle on the left. Carbon is in either dark green or gold. Nitrogen is in blue. Oxygen is in red. (F) Detailed view of the residues involving in the vestibule pocket in the antagonist-bound model of ECD-Asu-ACR-16. Functionally important amino acids which interact with the allosteric modulators bound in this region are labeled. (G) Detailed view of the top pocket relative to the location of the orthosteric binding site in the antagonist-bound model of ECD-Asu-ACR-16. Principal subunit is colored light pink, whereas the complementary subunit is colored yellow. The residues that contribute to the vestibule pocket are represented by sticks (principal side (+), purple) and highlighted inside the red dotted circle at the top. Carbon is in purple. Nitrogen is in blue. Oxygen is in red. (H) Detailed view of the residues involving in the top pocket in the antagonist-bound model of ECD-Asu-ACR-16. Functionally important amino acids which interact with the allosteric modulators bound in this region are labeled. (I) Detailed view of the two transmembrane allosteric binding sites in the antagonist-bound model of TID-Asu-ACR-16. Principal subunit is colored light pink, whereas the complementary subunit is colored yellow. The residues that contribute to the intersubunit site (principal side, pink; complementary side, orange) and intrasubunit site (principal side, purpleblue) are represented by sticks and highlighted inside the red dotted circle. Carbon is in either pink or orange or purpleblue. Nitrogen is in blue. Oxygen is in red. Sulfur is in yellow. (J) Detailed view of the residues involving in the two transmembrane allosteric binding sites in the antagonist-bound model of TID-Asu-ACR-16. Functionally important amino acids which interact with the allosteric modulators bound in this region are labeled.

Figure S3. Bar chart showing effects of the four hits on Asu-ACR-16. Results are expressed as maximum inhibition (mean ± S.E.M, %) of each plots in (Fig. 5A). The rank order series of inhibition based on maximum inhibition percentage for four hits is: SB-277011-A (96.07 ± 10.66%, n = 4) ≈ fmoc-1 (82.49 ± 4.74%, n = 4) ≈ fmoc-2 (80.34 ± 10.32%, n = 4) ≈ (+)-butaclamol Cl (79.53 ± 12.41%, n = 4) using unpaired t-test.

Figure S4. Sample traces showing the effects of four hits on the acetylcholine concentration-response relationships for Asu-ACR-16. Sample trace of acetylcholine concentration-response relationships in the absence of hits is depicted in (A) as a control. 3 μM of each of four hits are applied: (B) fmoc-2, (D) SB-277011, (F) fmoc-1, (H) (+)-butaclamol Cl, to compare the EC50 shifts and the maximum response reduction for four hits. 1 μM SB-277011-A (C), 1 μM (+)-butaclamol Cl (G) and 10 μM SB-277011-A (E) were tested to study the concentration effects on the mode of inhibition.

Figure S5. Ach concentration-response curves for Asu-ACR-16 in the absence of hits as a control (ach) and in the continual presence of 1 μM, 3 μM, 10 μM SB-277011-A, 1 μM, 3 μM (+)-butaclamol Cl.

Figure S6. Plot of average number of L4 C. elegans larvae with normal motility (A) vs. time (min) in the absence of drug (1% DMSO, control), presence of 30 μM SB-277011-A and 30 μM levamisole. Ten worms were used for each treatment, which was replicated by five times. Comparisons of locomotion were made between control and treated worms at each time point. *p < 0.05, **p < 0.01, ***p < 0.001. The recovery of normal motility was observed only in the larvae treated with 30 μM SB-277011-A within 20 min, but not in the larvae treated with 30 μM levamisole within 24 h.

Figure S7. Sequence and numbering of the full-length Asu-ACR-16 and its alignment with the human α7 nAChR subunit. Completely conserved residues (red background) and partially conserved residues (yellow background) are indicated. Secondary structures are shown schematically above the sequences. α1 represents α helix. β1-7 represent β strand. η1 represents 310 helix. The Cysteine loop labeled by green bars. Four transmembrane α helixes (M1, M2, M3 and M4) are labeled by blue coils. Residues in the orthosteric binding site are indicated by arrows (principal subunit, pink; complementary subunit, orange). Residues in four allosteric binding sites are highlighted by arrows (principal subunit of agonist sub-pocket, turquoise; complementary subunit of agonist sub-pocket, green; principal subunit of vestibule pocket, dark green; complementary subunit of vestibule pocket, gold; principal subunit of top pocket, purple; principal subunit of intersubunit transmembrane site: bright pink; complementary subunit of intersubunit site: brown; principal subunit of intrasubunit site: purpleblue).

Figure S8. Comparison of residues in the orthosteric binding site and five allosteric binding sites between Asu-ACR-16 and human α7 nAChR.
