# Supplementary material for: The Ascaris suum nicotinic receptor, ACR-16, as a drug target: Four novel negative allosteric modulators from virtual screening
Source: Int J Parasitol Drugs Drug Resist. 2016 Feb 10;6(1):60–73. doi: 10.1016/j.ijpddr.2016.02.001 (PMC4805779; doi:10.1016/j.ijpddr.2016.02.001)
Supplement: Supplementary file 1 [file mmc1.pptx]

## Slide 1
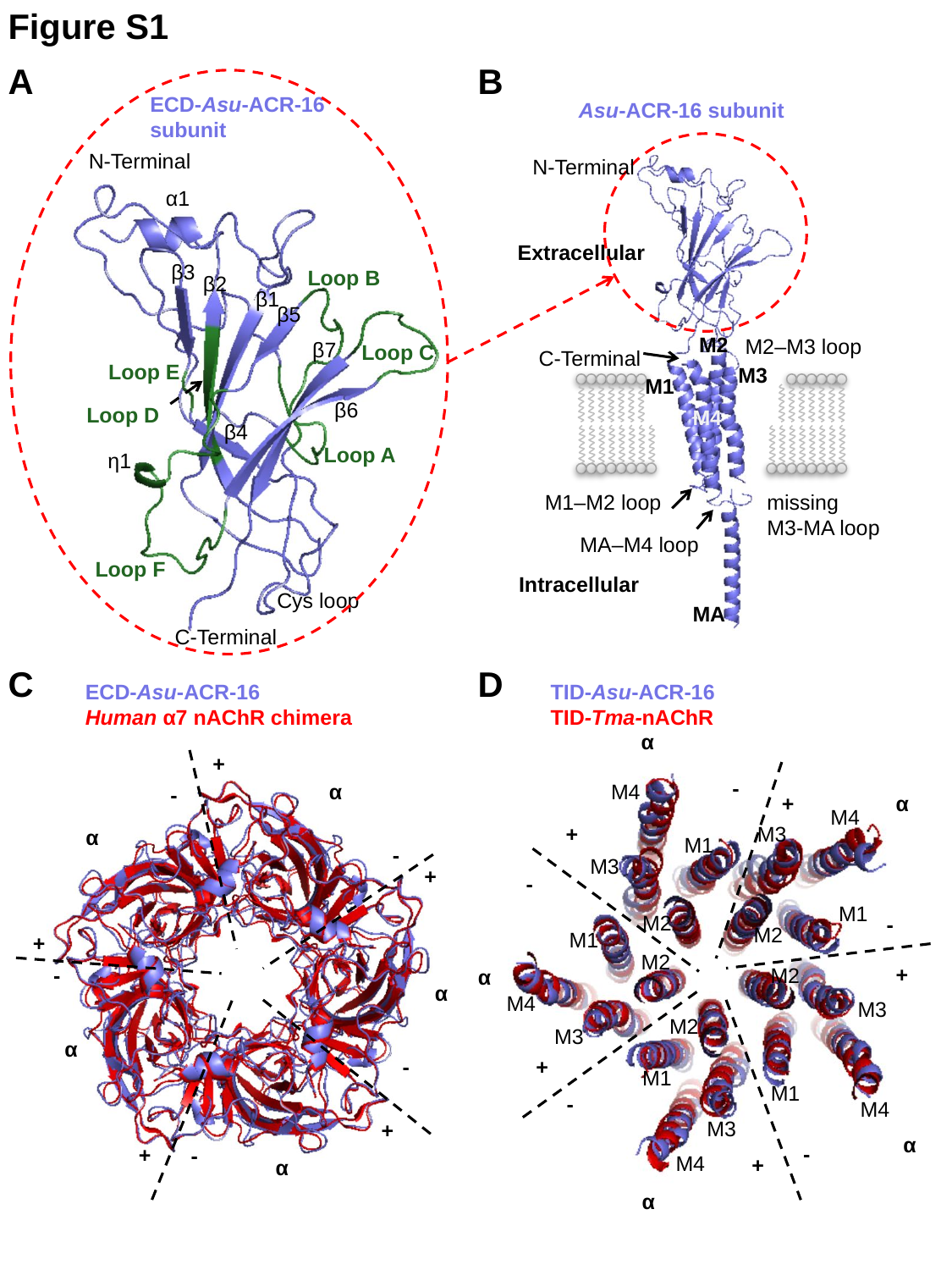

Figure S1
A
B
ECD-Asu-ACR-16
subunit
Asu-ACR-16 subunit
Extracellular
M2
M3
M1
M4
Intracellular
N-Terminal
α1
β3
β2
β1
β5
β7
β6
β4
Loop C
η1
Cys loop
C-Terminal
Loop B
Loop E
Loop D
Loop A
Loop F
N-Terminal
M2–M3 loop
C-Terminal
M1–M2 loop
missing
M3-MA loop
MA–M4 loop
MA
C
D
ECD-Asu-ACR-16
Human α7 nAChR chimera
+
α
-
α
-
+
+
-
α
α
-
+
+
-
α
TID-Asu-ACR-16
TID-Tma-nAChR
α
-
M4
+
α
M4
M3
+
M1
M3
-
M2
-
M1
M2
M1
M2
+
M2
α
M4
M3
M2
M3
+
M1
M1
-
M4
M3
α
-
M4
+
α
M1

## Slide 2
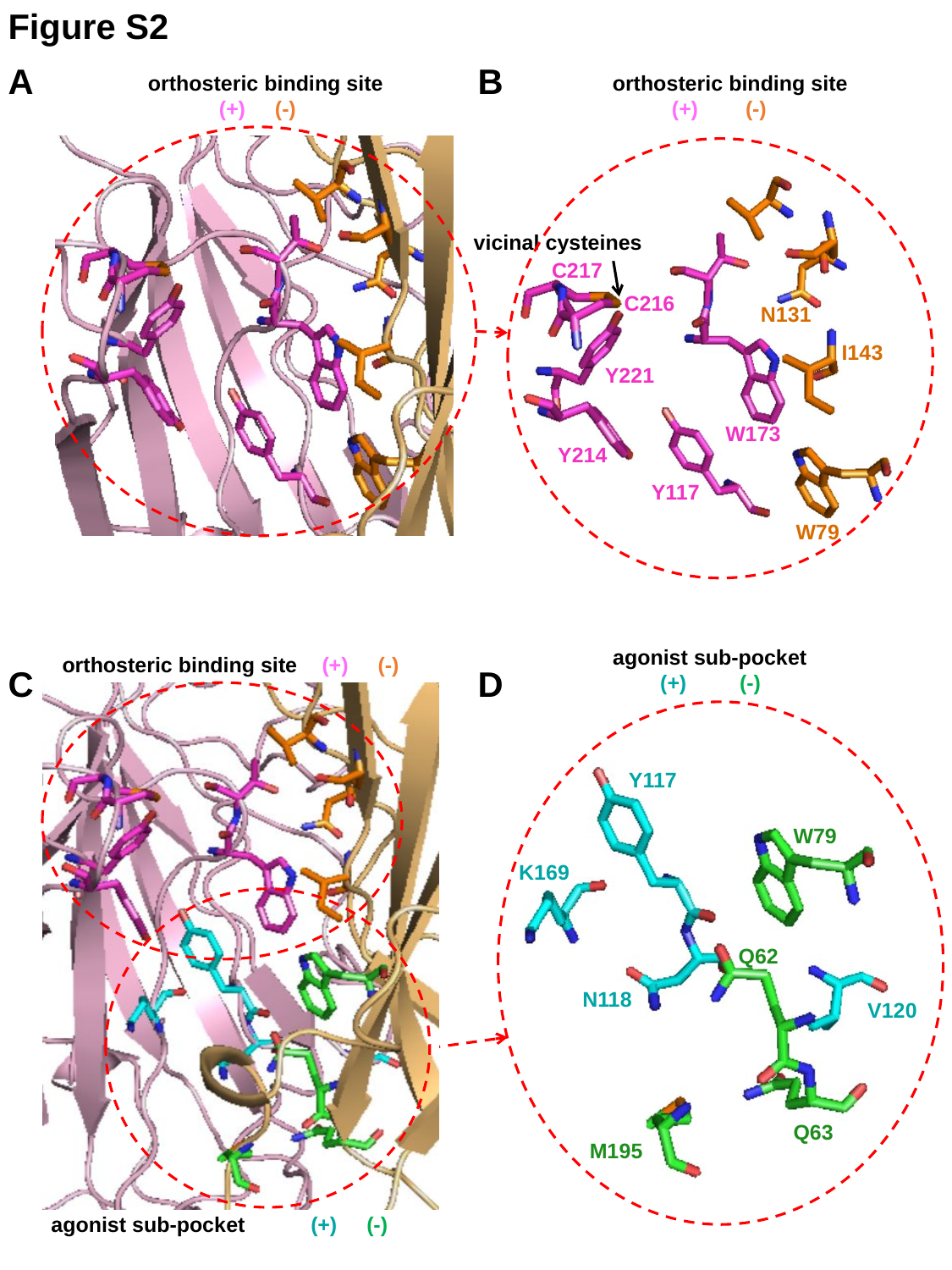

Figure S2
A
B
orthosteric binding site
 (+)	(-)
orthosteric binding site
 (+)	 (-)
vicinal cysteines
N131
I143
Y221
W173
Y214
Y117
W79
C217
C216
agonist sub-pocket
 (+) (-)
orthosteric binding site	 (+) (-)
agonist sub-pocket	 (+) (-)
C
D
Y117
W79
K169
Q62
N118
V120
Q63
M195

## Slide 3
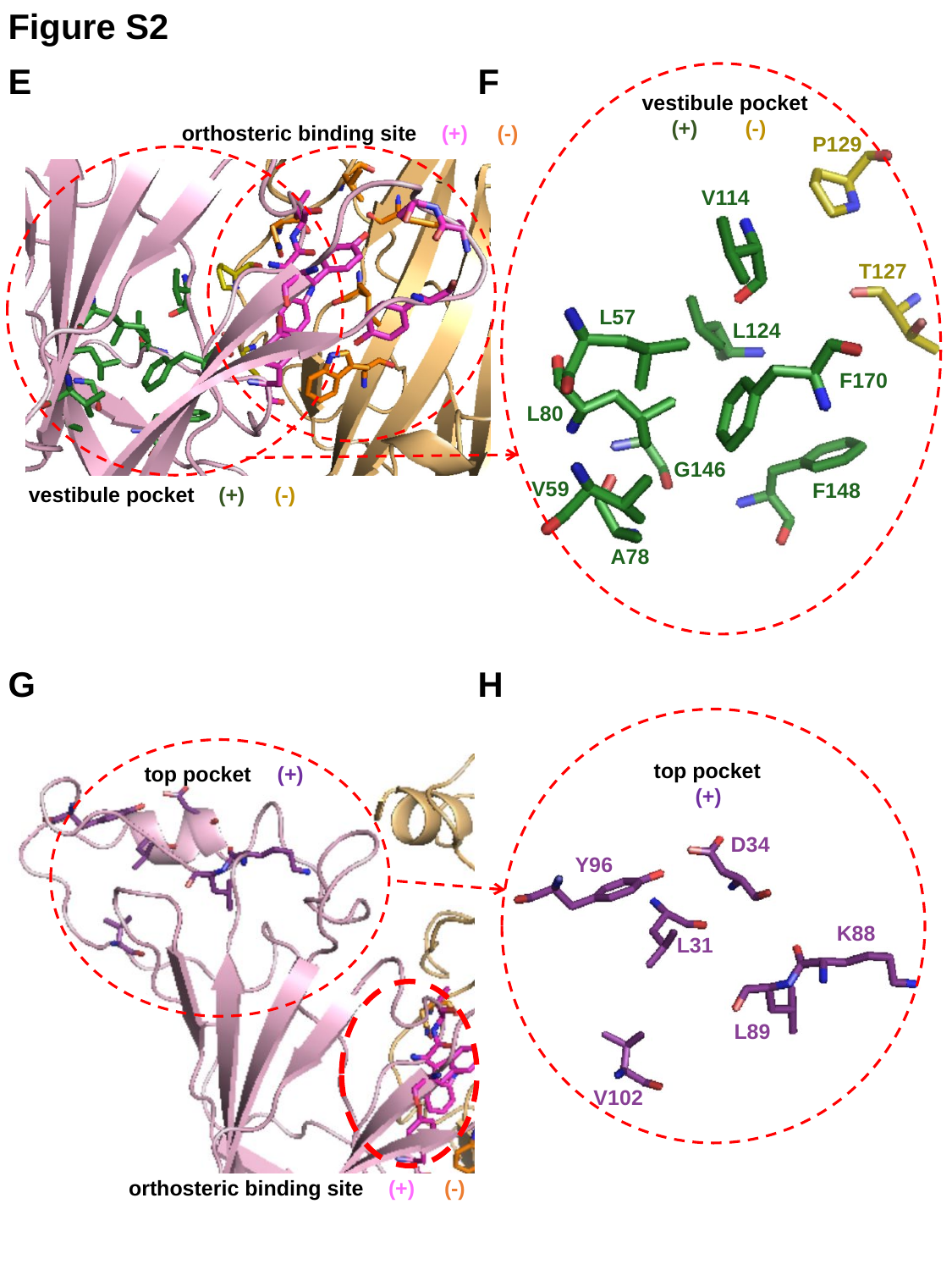

Figure S2
E
F
vestibule pocket
 (+) (-)
orthosteric binding site	 (+) (-)
vestibule pocket (+) (-)
P129
V114
T127
L57
L124
F170
L80
G146
V59
F148
A78
G
H
top pocket
 (+)
top pocket	 (+)
orthosteric binding site	 (+) (-)
D34
Y96
K88
L31
L89
V102

## Slide 4
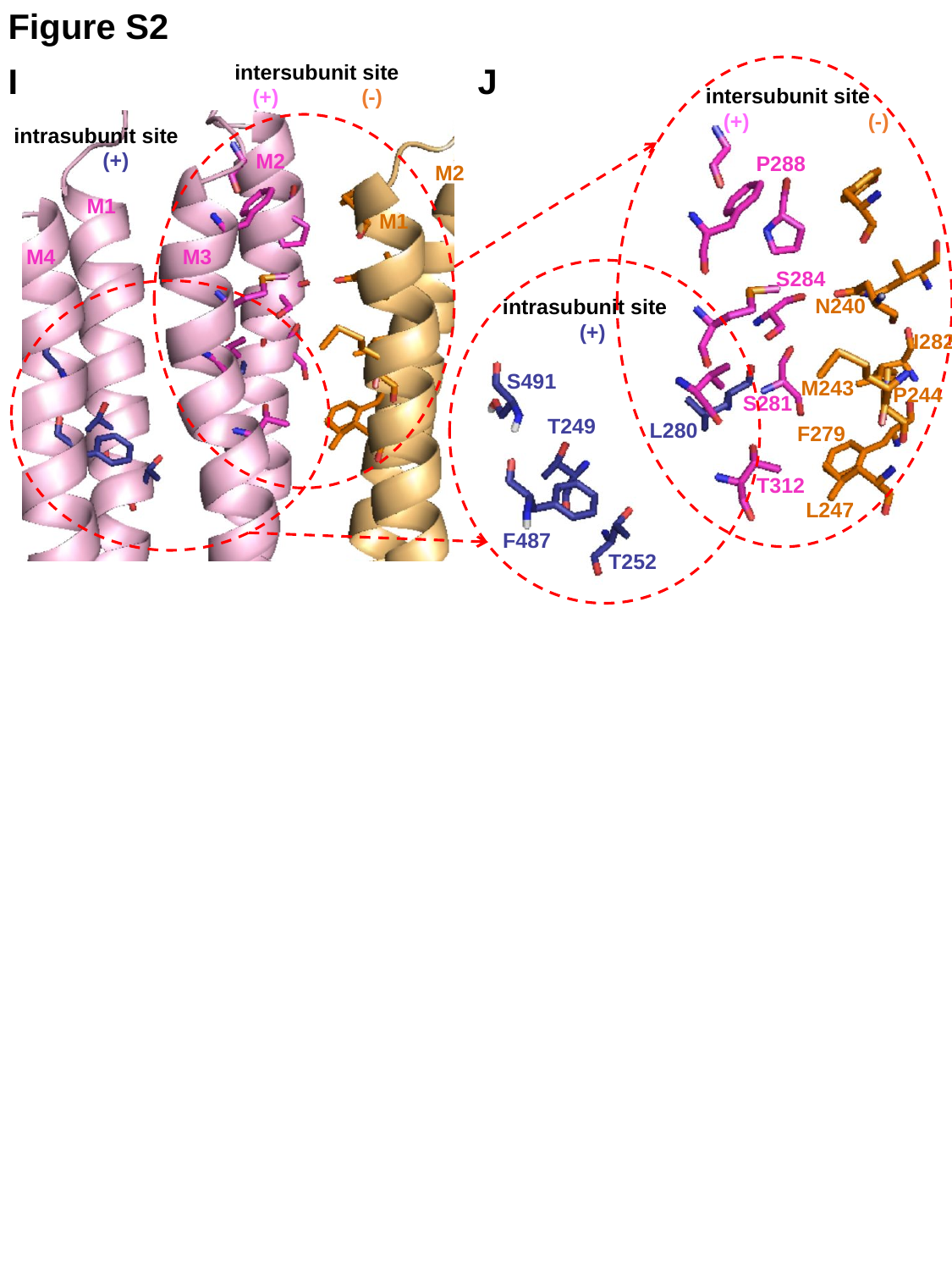

Figure S2
intersubunit site
 (+) (-)
I
J
intersubunit site
 (+)	 (-)
M2
M1
M3
M1
M4
M2
intrasubunit site
 (+)
P288
S284
N240
intrasubunit site
 (+)
I282
S491
M243
P244
S281
T249
L280
F279
T312
L247
F487
T252

## Slide 5
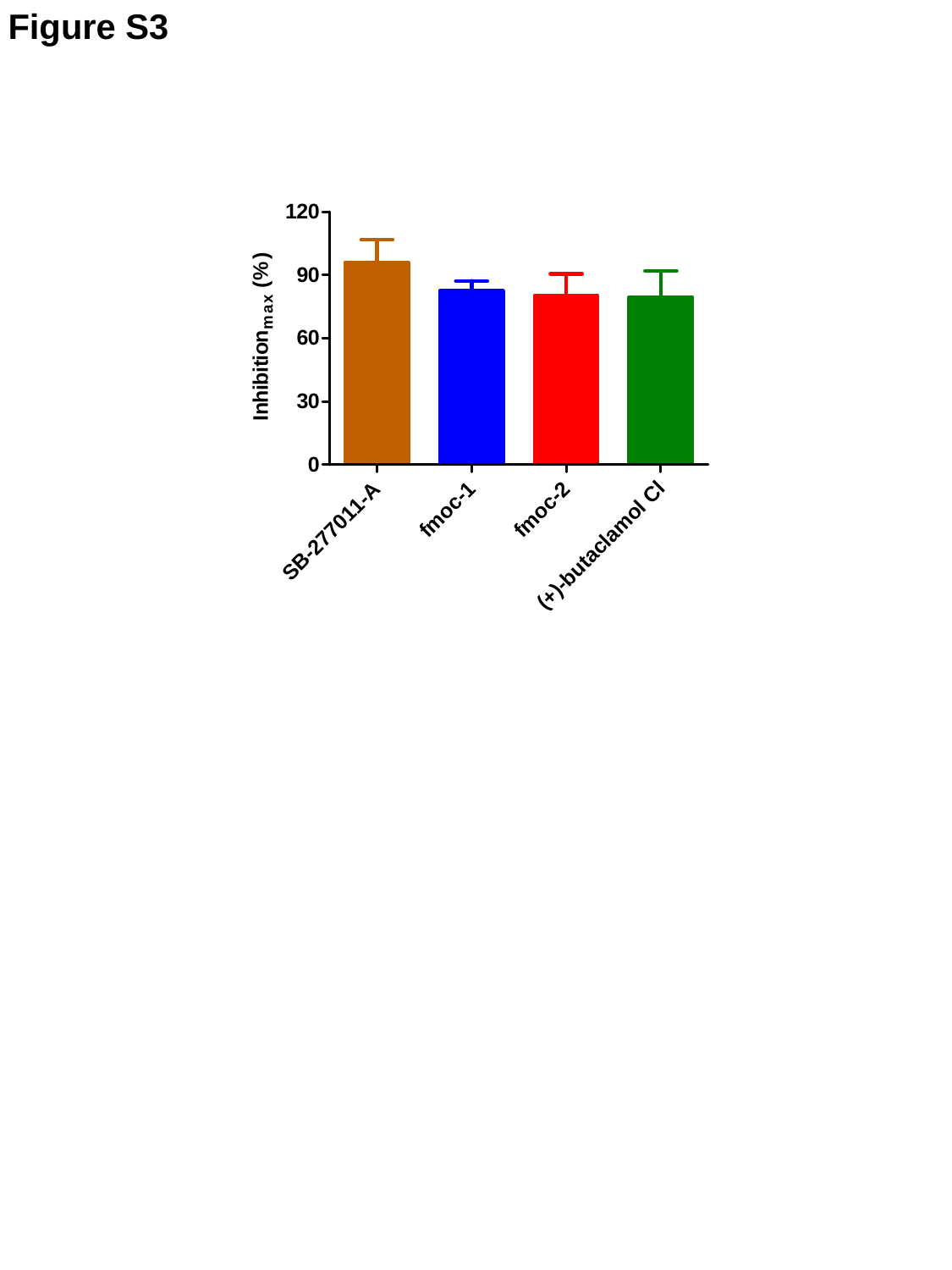

Figure S3

## Slide 6
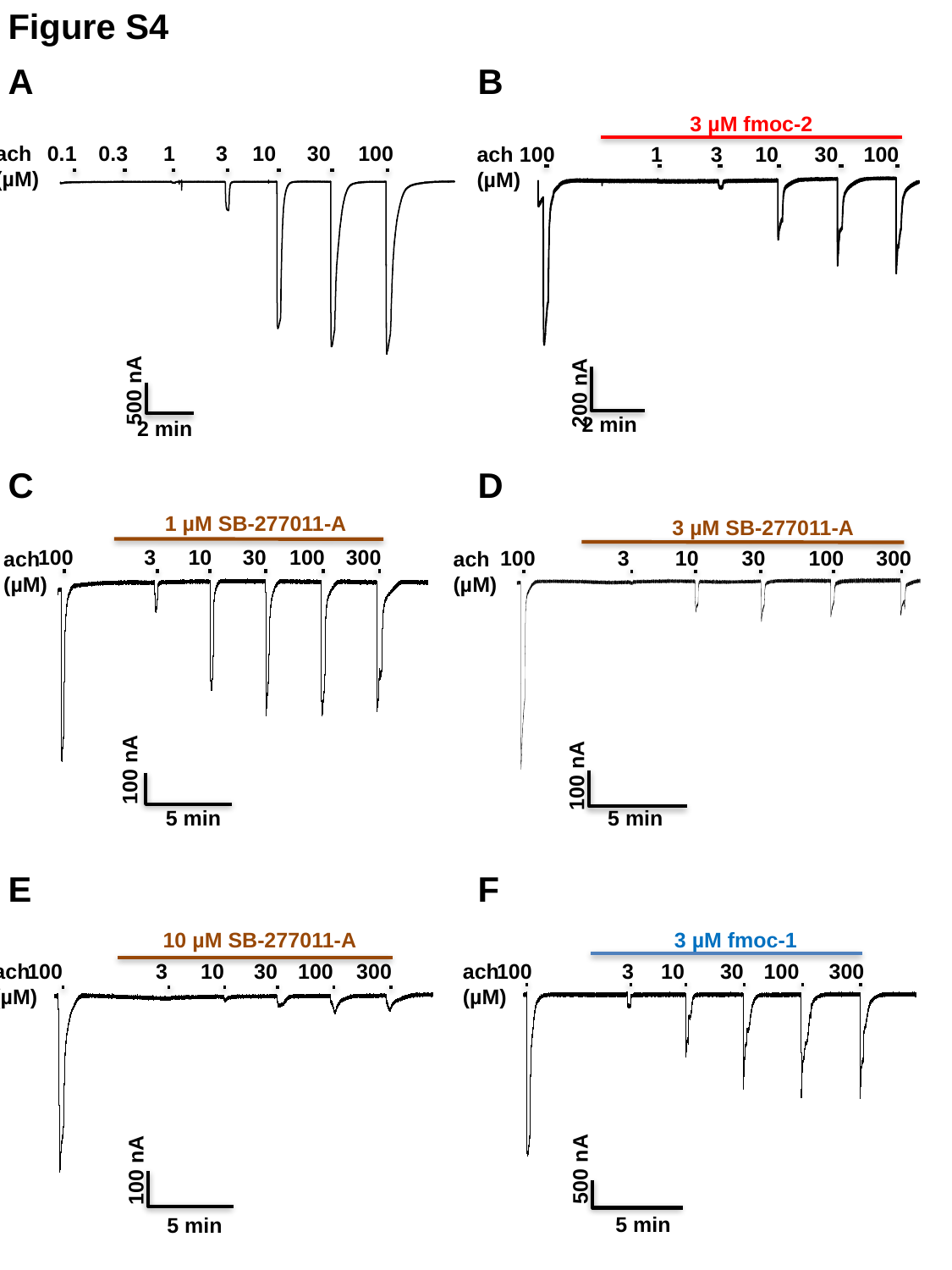

Figure S4
A
B
3 µM fmoc-2
ach
(µM)
100
1
3
10
30
100
200 nA
2 min
0.1
0.3
1
3
10
30
100
ach
(µM)
500 nA
2 min
C
D
1 µM SB-277011-A
100
3
10
30
100
300
ach
(µM)
100 nA
5 min
3 µM SB-277011-A
100
3
10
30
100
300
ach
(µM)
100 nA
5 min
E
F
10 µM SB-277011-A
ach
(µM)
100
3
10
30
100
300
100 nA
5 min
3 µM fmoc-1
3
ach
(µM)
100
10
30
100
300
500 nA
5 min

## Slide 7
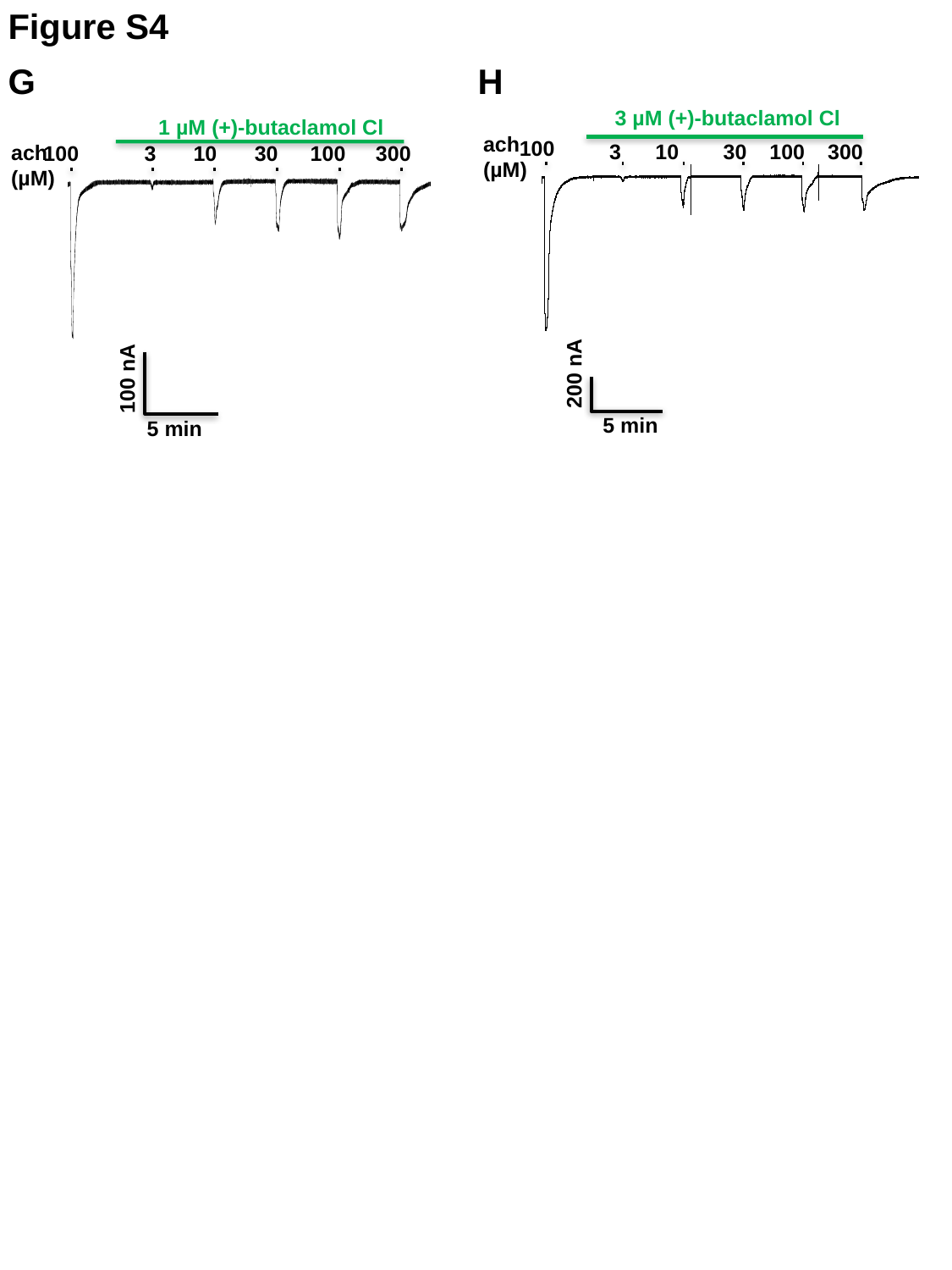

Figure S4
G
H
3 µM (+)-butaclamol Cl
ach
(µM)
100
3
10
30
100
300
200 nA
5 min
1 µM (+)-butaclamol Cl
ach
(µM)
100
3
10
30
100
300
100 nA
5 min

## Slide 8
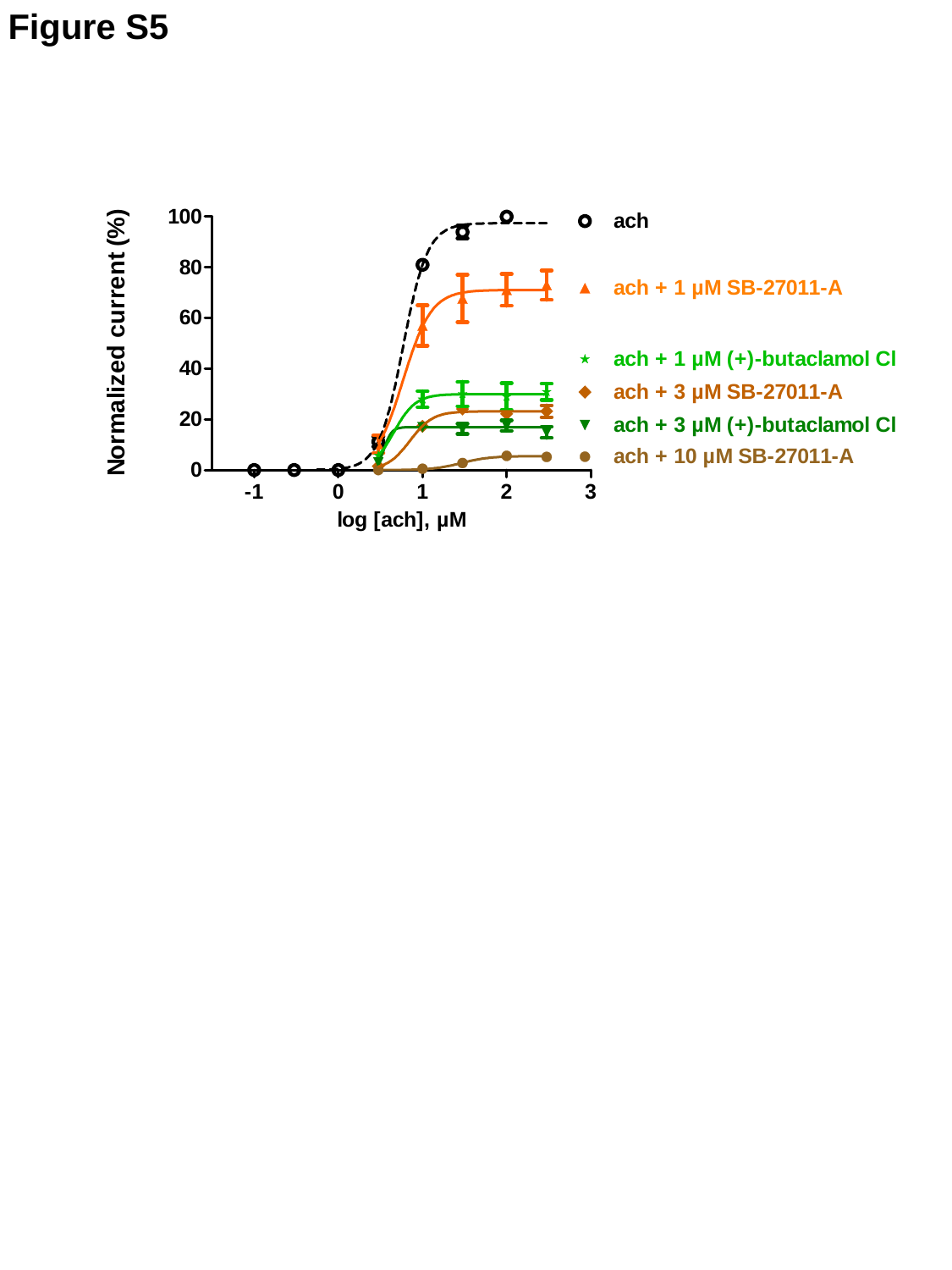

Figure S5

## Slide 9
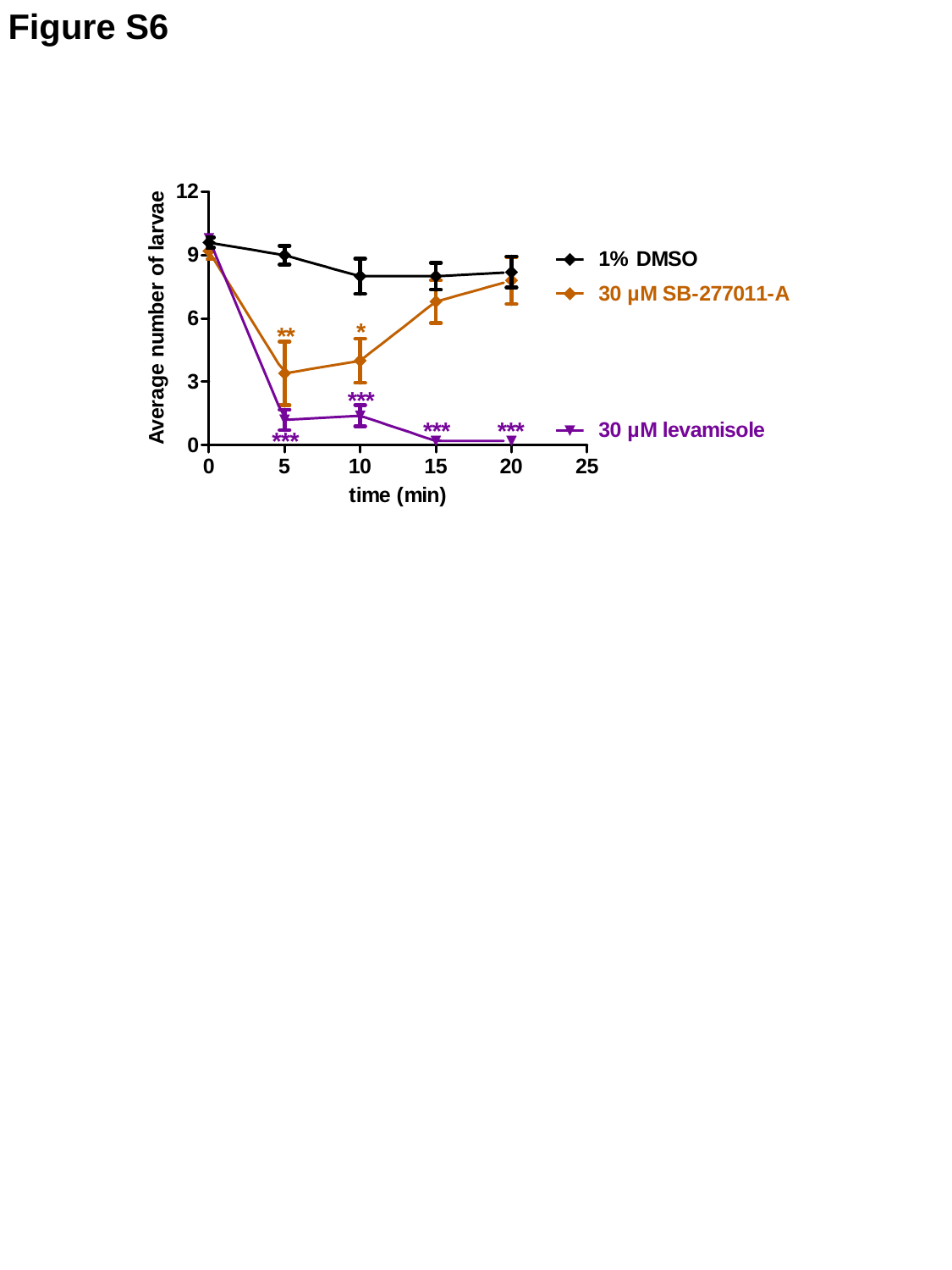

Figure S6

## Slide 10
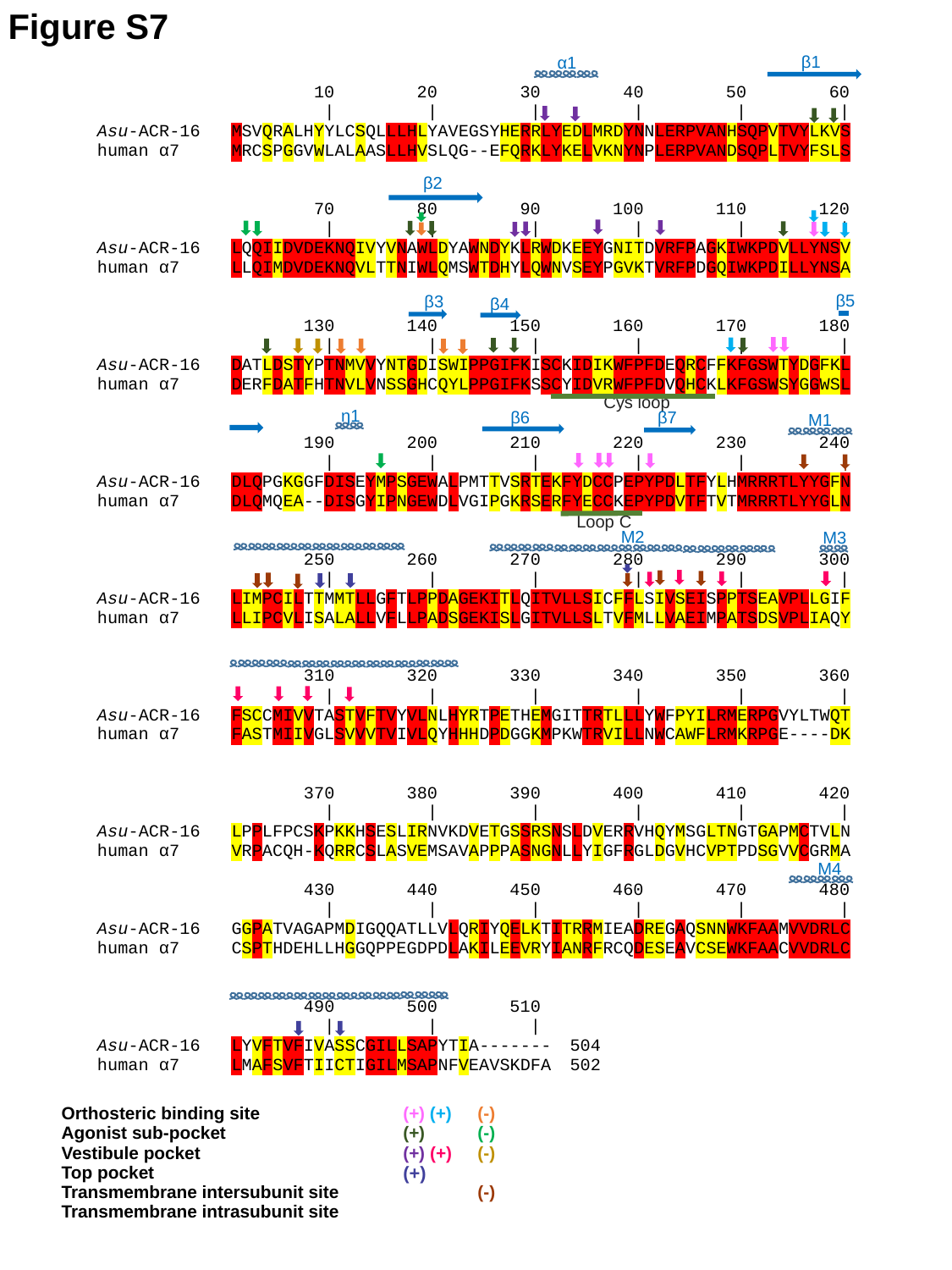

Figure S7
| Orthosteric binding site Agonist sub-pocket Vestibule pocket Top pocket Transmembrane intersubunit site Transmembrane intrasubunit site | (+) (+) (+) (+) (+) (+) | (-) (-) (-) (-) |
| --- | --- | --- |

## Slide 11
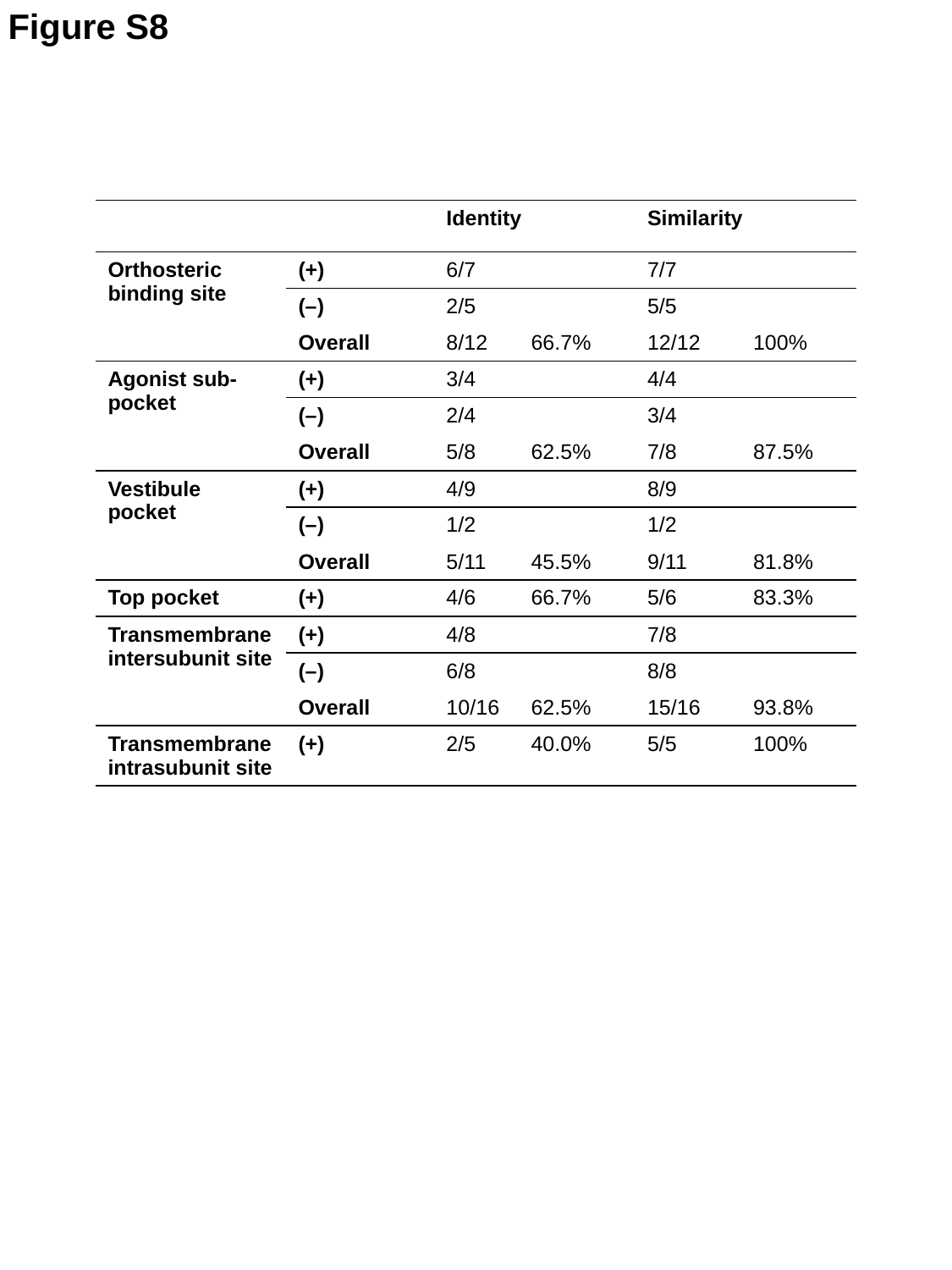

Figure S8
| | | Identity | | Similarity | |
| --- | --- | --- | --- | --- | --- |
| Orthosteric binding site | (+) | 6/7 | | 7/7 | |
| | (–) | 2/5 | | 5/5 | |
| | Overall | 8/12 | 66.7% | 12/12 | 100% |
| Agonist sub-pocket | (+) | 3/4 | | 4/4 | |
| | (–) | 2/4 | | 3/4 | |
| | Overall | 5/8 | 62.5% | 7/8 | 87.5% |
| Vestibule pocket | (+) | 4/9 | | 8/9 | |
| | (–) | 1/2 | | 1/2 | |
| | Overall | 5/11 | 45.5% | 9/11 | 81.8% |
| Top pocket | (+) | 4/6 | 66.7% | 5/6 | 83.3% |
| Transmembrane intersubunit site | (+) | 4/8 | | 7/8 | |
| | (–) | 6/8 | | 8/8 | |
| | Overall | 10/16 | 62.5% | 15/16 | 93.8% |
| Transmembrane intrasubunit site | (+) | 2/5 | 40.0% | 5/5 | 100% |
